# Past, present, and future of electrical impedance tomography and myography for medical applications: a scoping review

**DOI:** 10.3389/fbioe.2024.1486789

**Published:** 2024-12-11

**Authors:** Lea Youssef Baby, Ryan Sam Bedran, Antonio Doumit, Rima H. El Hassan, Noel Maalouf

**Affiliations:** ^1^ Electrical and Computer Engineering Department, Lebanese American University, Byblos, Lebanon; ^2^ Biomedial Engineering Department, SciNeurotech Lab, Polytechnique Montréal, Montréal, QC, Canada

**Keywords:** biosensors, electrical impedance, electrical impedance myography, electrical impedance tomography, electrical impedance for medical applications

## Abstract

This scoping review summarizes two emerging electrical impedance technologies: electrical impedance myography (EIM) and electrical impedance tomography (EIT). These methods involve injecting a current into tissue and recording the response at different frequencies to understand tissue properties. The review discusses basic methods and trends, particularly the use of electrodes: EIM uses electrodes for either injection or recording, while EIT uses them for both. Ag/AgCl electrodes are prevalent, and current injection is preferred over voltage injection due to better resistance to electrode wear and impedance changes. Advances in digital processing and integrated circuits have shifted EIM and EIT toward digital acquisition, using voltage-controlled current sources (VCCSs) that support multiple frequencies. The review details powerful processing algorithms and reconstruction tools for EIT and EIM, examining their strengths and weaknesses. It also summarizes commercial devices and clinical applications: EIT is effective for detecting cancerous tissue and monitoring pulmonary issues, while EIM is used for neuromuscular disease detection and monitoring. The role of machine learning and deep learning in advancing diagnosis, treatment planning, and monitoring is highlighted. This review provides a roadmap for researchers on device evolution, algorithms, reconstruction tools, and datasets, offering clinicians and researchers information on commercial devices and clinical studies for effective use and innovative research.

## 1 Introduction

Scientists have been studying the bio-electrical properties of living tissues using several techniques to better understand healthy tissues and facilitate the diagnosis of pathological conditions. One of the earliest instances of electrical resistivity analysis is the study by Stefanesco et al. (1930), which dates back to nearly a century. In the early stages, the term resistivity was adopted instead of impedance. Another electrical impedance analysis technique was introduced by Fatt (1964). The technique is based on the electrical and frequency-dependent impedance changes that occur in various tissues when subjected to excitation (internal or external). Electrical impedance technologies involve injecting current/voltage into the target being studied, recording the response, and analyzing the change in properties (impedance, conductivity, and permittivity) between the two signals through a processing unit.

Impedance analysis is considered a powerful imaging and diagnostic tool. The technology can be used to assess muscle health, identify diseases, create images of target organs, and provide real-time monitoring for critical patients (Rutkove, 2009; Cebrián-Ponce et al., 2021; Shi et al., 2021; Li et al., 2022). Electrical impedance analysis—an effective, non-invasive, and safe tool—has become an interesting technology for several research groups worldwide. Moreover, the field of electrical impedance technologies has witnessed substantial growth over the past decade, reflecting an increasing interest in non-invasive diagnostic and monitoring tools for various medical applications. In electrical impedance tomography (EIT), the number of publications related to clinical applications has increased steadily. A bibliometric study indicates that from 2011 to 2021, the annual output of EIT-related research articles nearly doubled, with significant contributions from research institutions in the United States, United Kingdom, Germany, and China (Tan et al., 2023). This is validated by cross-checking the data with the Web of Science database (Clarivate, 2024). Research topics spanned clinical applications in lung monitoring, cardiac assessment, and neural activity, indicating the versatility and expanding role of EIT in real-time physiological monitoring (Qin et al., 2022). Likewise, electrical impedance myography (EIM) has shown a marked increase in publications, particularly concerning its applications in neuromuscular and musculoskeletal health. Bibliometric analyses identify a growth trajectory that aligns with advancements in wearable bioimpedance devices, which facilitate EIM’s application in both clinical and at-home settings. The total number of publications in EIM has been projected to continuously increase as research increasingly focuses on algorithmic enhancements and integration with machine learning for better disease tracking and diagnostic accuracy (Li et al., 2022). This growth in EIT and EIM publications highlights the substantial advancements in algorithm development, hardware optimization, and clinical validation studies, making these technologies essential tools in modern diagnostic imaging and monitoring. Several published review papers cover multiple areas of bio-impedance measurement and imaging. Bayford (2006) reviewed the evolution of EIT as a clinical tool and covered the hardware and software development of EIT systems. McEwan et al. (2007) reviewed the instrumentation of EIT systems that are capable of injecting and analyzing signals at several frequencies instead of a single frequency. Rutkove (2009) surveyed another major application of impedance analysis, EIM, and explained how electrical activity in tissues and its variations in diseased tissues can be detected with EIM, leading to the diagnosis of neuro-muscular diseases. Rutkove further published another survey with Sanchez focusing on the studies that used EIM to assess neuromuscular diseases and studied their progression and response to treatments (Sanchez and Rutkove, 2017b). Finally, Cebrián-Ponce et al. (2021) systematically reviewed the studies on EIM applications in health and physical exercise. Shi et al. (2021) focused their review on the progress of EIT hardware and software for lung diseases, while Li et al. (2022) published a systematic review of EIT for clinical lung monitoring. Another limitation to the most recent survey papers is that they are either software-focused (Tan et al., 2023) or hardware-oriented (Qin et al., 2022). The main EIT and EIM survey papers published in the past two decades are summarized in Table 1. These papers only cover specific niche areas within the broad modalities.

**TABLE 1:** Summary of review papers.

Title	Date	Reference
Bioimpedance tomography (electrical impedance tomography)	29 March 29 2006	Bayford (2006)
A review of errors in multi-frequency EIT instrumentation	26 June 2007	McEwan et al. (2007)
Electrical impedance myography: Background, current state, and future directions	18 September 2009	Rutkove (2009)
Present uses, future applications, and technical underpinnings of electrical impedance myography	20 September 2017	Sanchez and Rutkove (2017b)
Electrical impedance myography in health and physical exercise: A systematic review and future perspectives	14 September 2021	Cebrián-Ponce et al. (2021)
The research progress of electrical impedance tomography for lung monitoring	October 2021	Shi et al. (2021)
Emerging trends and hot spots of electrical impedance tomography applications in clinical lung monitoring	31 January 2022	Li et al. (2022)
Characteristics and topic trends on electrical impedance tomography hardware publications	13 October 2022	Qin et al. (2022)
Research trends and hot spots of medical electrical impedance tomography algorithms: A bibliometric analysis from 1987 to 2021	30 November 2023	Tan et al. (2023)

Given that EIT and EIM remain very similar modalities with different endpoints, we decided to review the evolution of both modalities while addressing the limitations to existing survey papers. The main goal of this review paper is twofold: (1) to present a general overview of the state of the art in EIT and EIM technologies for researchers entering this field for the first time and (2) to highlight possible areas of contribution that will improve the use of EIT and EIM for monitoring, diagnosis, and assistance.

This survey paper contributes to answering five main questions related to EIT and EIM.

1. What are the main components that constitute any EIT or EIM system?
2. What are the specific considerations that must be accounted for when using EIT and EIM for different applications?
3. How is the obtained data processed for accurate analysis?
4. Which diseases have been targeted by EIT and EIM and what are the possible ground-breaking works that these technologies are capable of achieving in the near future for the diagnosis and therapy of other diseases?
5. Can we use EIT and EIM for predicting diseases, tumor growths, and muscular fatigue/injuries? What are the currently available deep learning models and datasets? Do these models rely only on EIT/EIM or a fusion with other modalities?

**FIGURE 1:**
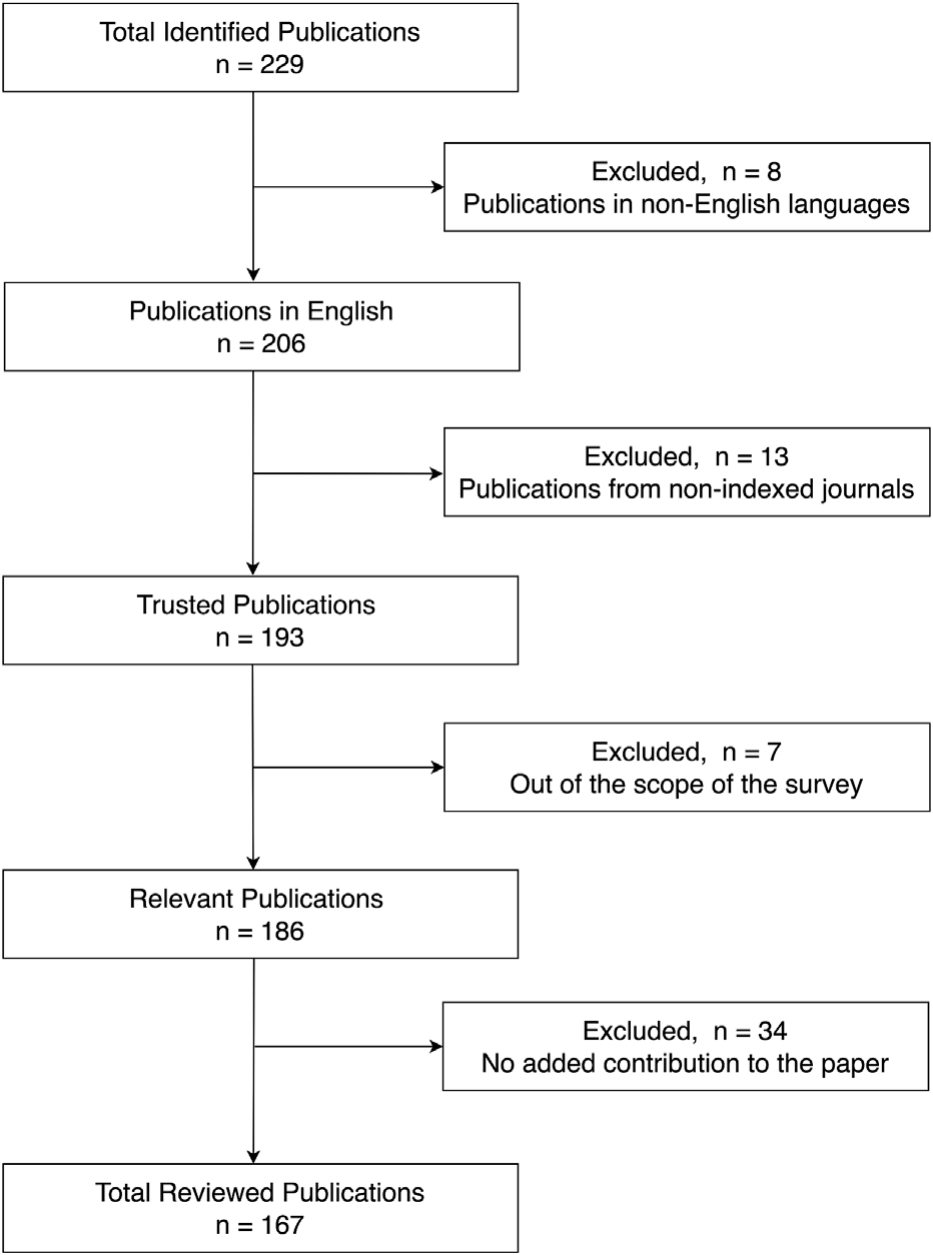
Article selection breakdown.

This review paper covers articles from IEEE Xplore, ScienceDirect, Elsevier, PubMed, IOPscience, Frontiers, and SAGE databases. The following search terms were used in the queries: (1) “Electrical Impedance Tomography,” (2) “Electrical Impedance Myography,” (3) “Magnetic Resonance Electrical Impedance Tomography,” (4) “lung diseases detection using Electrical Impedance Tomography,” (5) “brain imaging using Electrical Impedance Tomography,” (6) “Electrical Impedance Tomography hardware,” (7) “bioimpedance,” (8) “Electrical Impedance Tomography algorithms,” (9) “Electrical Impedance Tomography forward problem,” and (10) “Electrical Impedance Tomography inverse problem.” We initially collected 229 articles, of which 167 were referenced in this paper. Articles were filtered out based on a multitude of reasons, mainly articles that lacked contribution to EIT on the hardware level, articles that were from non-indexed journals, and articles that were out of the scope of this paper. We also excluded articles that were already referenced by other included papers. After completing the selection process, a total of 152 relevant publications were reviewed, as shown in Figure 1.

The remainder of the article is organized as follows: Section 2 presents an overview of EIT and EIM modalities and the corresponding electrode configurations. Advancements in EIT and EIM systems design are reviewed in Section 3, showing the evolution of the devices over time, the major design components, and available commercial devices in the market. Section 4 presents the reconstruction and signal processing techniques used in EIT and EIM, highlighting state-of-the-art algorithms. Sections 5, 6 cover the advancements in EIT and EIM, respectively, in the diagnosis, monitoring, and treatment planning of diseases. Section 7 explores the applications of cell-substrate impedance in regenerative medicine and cardiology. The role of deep learning techniques in EIM is reviewed in Section 8, where the relevant publicly available datasets are presented. Section 9 discusses the findings of surveying the state of the art in EIT and EIM and highlights potential areas of research contributions to the aforementioned fields, while Section 10 concludes the review paper.

## 2 Electrical impedance methods—monitoring and imaging

Over the past decade, the focus in the bioelectronics field has shifted toward diagnosing and analyzing different organs and body parts using non-invasive techniques for early disease diagnosis. The two most notable areas of focus have been EIT and EIM. Both technologies are non-invasive techniques used for assessing the health and function of organs/muscles by extracting information from electrical recordings—either through imaging or electrical properties of tissues. These techniques work by injecting electrical signals into target tissues/organs and recording responses. Differences in electrical properties between injected and recorded signals can be associated with muscle health and functioning. These differences are either processed using reconstruction algorithms to create medical images or compared to assess the health and activity of tissues. EIT has seen a major rise over the past 2 years, which is mostly in response to the COVID-19 pandemic, which has overwhelmed healthcare systems around the world and thus driven researchers into developing this technology further (Pennati et al., 2023). EIM is mostly used for the assessment of neuromuscular diseases and presents many advantages, such as being non-invasive and requiring minimal cooperation from subjects, which results in accurate and repeatable data. Both EIT and EIM can be used for real-time applications. However, since this paper focuses on human body-related applications, this section provides a global overview of the procedures followed to apply both techniques.

### 2.1 Overview of EIT and EIM modalities

EIT is an imaging technique used to visualize the tissue’s internal electrical properties and is widely employed in various applications such as detecting and monitoring lung diseases, cancerous tissues, and neural and brain activities, as well as evaluating perfusion and cardiac function (Adam and Ammaiappan, 2021). Measurements of the EIT systems are normally taken at electrodes placed on the surface of the body. The electrodes used can be polarized (stainless steel, conductive fabrics, and rubbers) or non-polarized (ECG-type silver/silver chloride (Ag/AgCl) electrodes) (Bayford, 2006). EIT is a highly sensitive technology where slight electrode movement may have major effects on the signal quality. To prevent motion artifacts, electrodes are integrated into a belt or harness and fixed around target organs, and gels and liquids are applied at the electrode–tissue interface (Zamora-Arellano et al., 2020). However, the quality of the data measured decreases as the measurement time is extended, which is due to the drying of electrodes. It can also be affected by motion artifacts and changes in the postures of the human or animal subjects under study. The applied waveform is mostly either sinusoidal or approximated by a square wave, with patterns to be discussed later. Injected waveforms can vary in intensity, frequency, or nature (voltage or current injection) but are always limited to the “electrical safety considerations,” which denote the maximum stimulation current over all the electrodes into the body. Safe currents are limited to a maximum of 10 mA at a frequency of 100 kHz (Lionheart et al., 2001). Finally, the obtained data need to be processed and analyzed to calculate “application-relevant” images and measures since it is virtually impossible to give relevant clinical information based on raw data (Adler and Boyle, 2017). Similar to EIT, EIM involves placing electrodes on the surface of the body, specifically across the muscle or muscle group of interest. However, fewer electrodes are usually needed for EIM when compared to EIT, with 4, 8, or 16 electrodes often placed to minimize “electrode polarization” and, therefore, conserve the signal quality. The choice of the electrode material varies with the application and the muscle of interest. The most commonly used ones are the Ag/AgCl electrodes; however, dry electrodes, metal electrodes, and textile dry electrodes are also used. The obtained data are processed and analyzed to extract features necessary to diagnose diseases or assess muscle health. Several factors affect injection and recording patterns, as well as electrode numbers in both EIT and EIM. First, image reconstruction in EIT requires a greater amount of data than myography. Overlapping-dependent recordings (explained in further sections) resulting from different injection and recording patterns limit the fidelity of reconstructed images and thus the need for a higher number of electrodes in EIT. Second, the nature of recorded signals, being mixed summation potentials from several neighboring fibers, affects the number of independent signals collected and the processing tools to be used (Zamora-Arellano et al., 2020; Shiffman et al., 2008). Finally, the configuration of electrodes plays a major role in the system’s hardware design to avoid interference and reduce noise; therefore, we will first discuss the available electrode configurations accompanied by injection and recording patterns before introducing the hardware components of the system.

### 2.2 Electrode configurations (injection and recording patterns)

**FIGURE 2:**
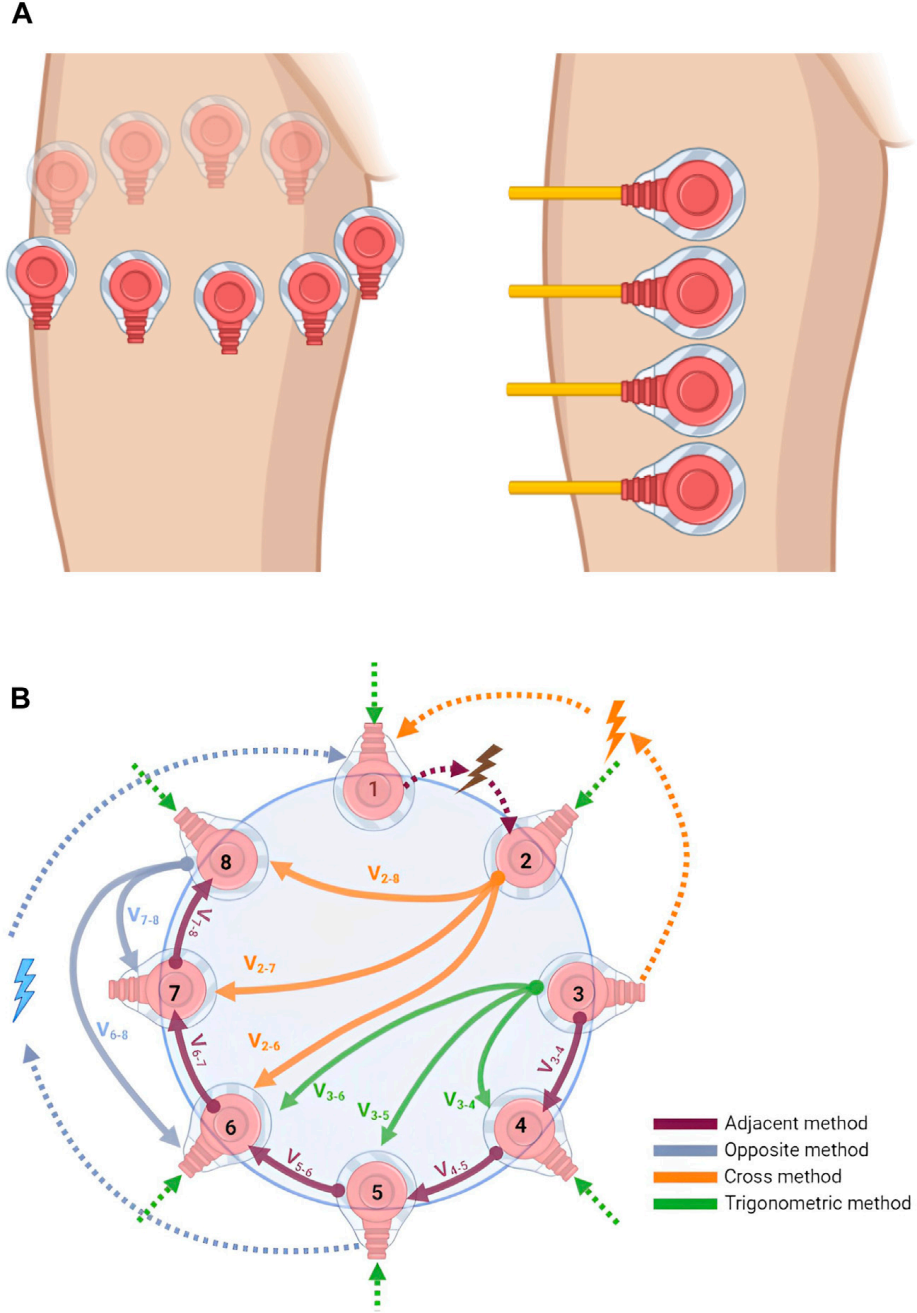
**(A)** Electrode placement configuration: in EIT (left), electrodes are placed around the area to be imaged, usually in a circular pattern, while in EIM (right), electrodes are placed linearly at the target muscle to be analyzed and studied. This image was produced using BioRender. **(B)** Electrode configurations for injection and recording pattern. The figure shows the adjacent, opposite, cross, and trigonometric injection/recording methods in an 8-electrode EIT system. Dotted arrows are for injection, other arrows are for recordings, and subscripts represent electrode numbers. This image was produced using BioRender.

Different configurations have been implemented for injecting and recording electrical signals. Early EIM systems were composed of only a few electrodes with non-varying positions, which were used for both stimulating and recording electrical signals (Rutkove, 2009). Later, the four-electrode configuration became the standard, which is characterized by the placement of the four electrodes over the muscle of interest in a linear disposition (Figure 2A-right). It should be noted that the sensitivity of the measurements is affected by the surrounding tissues, including the skin, fat, and bone. The injected signal is applied at the outer electrodes, and the produced response is then measured by the inner electrodes as an alternating voltage (Sanchez and Rutkove, 2017a). Higher electrode number systems are also available for EIM, such as 8- and 16-electrode configurations. In some eight-electrode systems, two electrodes are reserved for injection, while the remaining six, placed linearly adjacent to each other, are reserved for recording. This method requires careful placement of electrodes since the injection electrodes are far from the recording ones and the variation in spacing is critical to the obtained data (Rutkove et al., 2005).

As mentioned earlier, fewer electrodes are required for EIM. For EIT, studies usually opt for either 16, 32, or 64 electrodes. The number of electrodes depends on the target accuracy required and the size of the devices developed. Electrodes are usually placed around the target organ, as shown in Figure 2A (left). There are four common EIT injection methods, the adjacent or neighboring method, the opposite method, the cross method, and the adaptive or trigonometric method (Bera and Nagaraju, 2012). Figure 2B shows the differences between the four. In the adjacent method, as suggested by Brown and Seagar (1987), the current is injected into adjacent electrodes and the potential difference can be measured between adjacent electrode pairs. For example, if the current is injected at electrodes (E) $E_{x}$ and $E_{x+1}$ , then voltages are recorded from $E_{x+2}$ and $E_{x+3}$ , then from $E_{x+3}$ and $E_{x+4}$ , and so on. The process is then repeated with current injected at another pair and voltages recorded. Therefore, an n-electrode system will record n−3 recordings for each injection and, thus, a total of n(n−3) recordings; yet only half of these recordings are independent due to the identical repeated boundary measurements.

In the cross method, the system should have two injection electrodes and one reference electrode (Hua, 1987). First, in an n-electrode system, electrode 1 is dedicated for reference and electrodes n and 2 for injection; then n−3 recordings are taken, one from each electrode, with electrode 1 being the reference. Then, the process is repeated with all other odd-numbered electrodes, injecting between n and 4, 6, 8, …, while recordings are taken from the remaining electrodes, with electrode 1 still as the reference. This will result in (n/2−1)(n−3) recordings. The same procedure is repeated with electrode 2 as the reference and injections made between electrodes 3 and the remaining odd-numbered electrodes (5, 7, 9, 11 …), which also results in (n/2−1)(n−3) recordings. Note that not all of the recorded signals are independent. For instance, the potential between electrodes 3 and 1 will be recorded multiple times, depending on the injection site.

The opposite method involves placing the injection electrodes at opposite locations, between electrodes x and n/2+x (in an n-electrode system), and recording from all other electrodes with electrode x+1 as the reference electrode (Hua, 1987). This will result in n−3 recordings, which are repeated n times, leading to a total of n(n−3) recordings, of which only half are independent. In the adaptive or trigonometric method, the current is defined by trigonometric functions, hence the name. Current is injected through all electrodes but with different current distributions. The distributions should be homogeneous, and the injected current is a multiple of sin⁡θ or cos⁡θ . Recordings are obtained from n−1 electrodes with respect to a reference, and the distribution is then rotated by 360/n degrees. The recordings are repeated n/2 times. A total of (n/2)(n−1) independent recordings is obtained using this method. Figure 2B shows the difference in the mentioned configurations.

Table 2 shows the pros and cons of the four previously mentioned electrode configuration methods (Holder, 2005). The main characteristics that form the basis for comparison are implementation complexity and degree of sensitivity to central and boundary changes in the readings. Moreover, if the same number of electrodes is used across all methods (for example, 16 electrodes), both the adjacent and opposite methods will have the highest number of independent measurements (208 in this example), compared to a significantly lower value for the trigonometric method (120 measurements), and the lowest (91 measurements) for the cross method.

**TABLE 2:** Comparison of electrode configuration methods.

Method	Pros	Cons
Adjacent	- Simplest implementation - Very sensitive to changes at the boundary	- Insensitive to changes in central areas or bulk of targets
Opposite	- More sensitive to central changes than the adjacent method	- More complex electrode configuration - Less sensitive to boundary changes than the adjacent method
Cross	- Better sensitivity in non-peripheral regions	- Least used - Poor sensitivity at the boundary
Trigonometric	- Enhanced sensitivity distribution compared to the adjacent and opposite methods	- Complex hardware since each electrode needs an independent current driver

**FIGURE 3:**
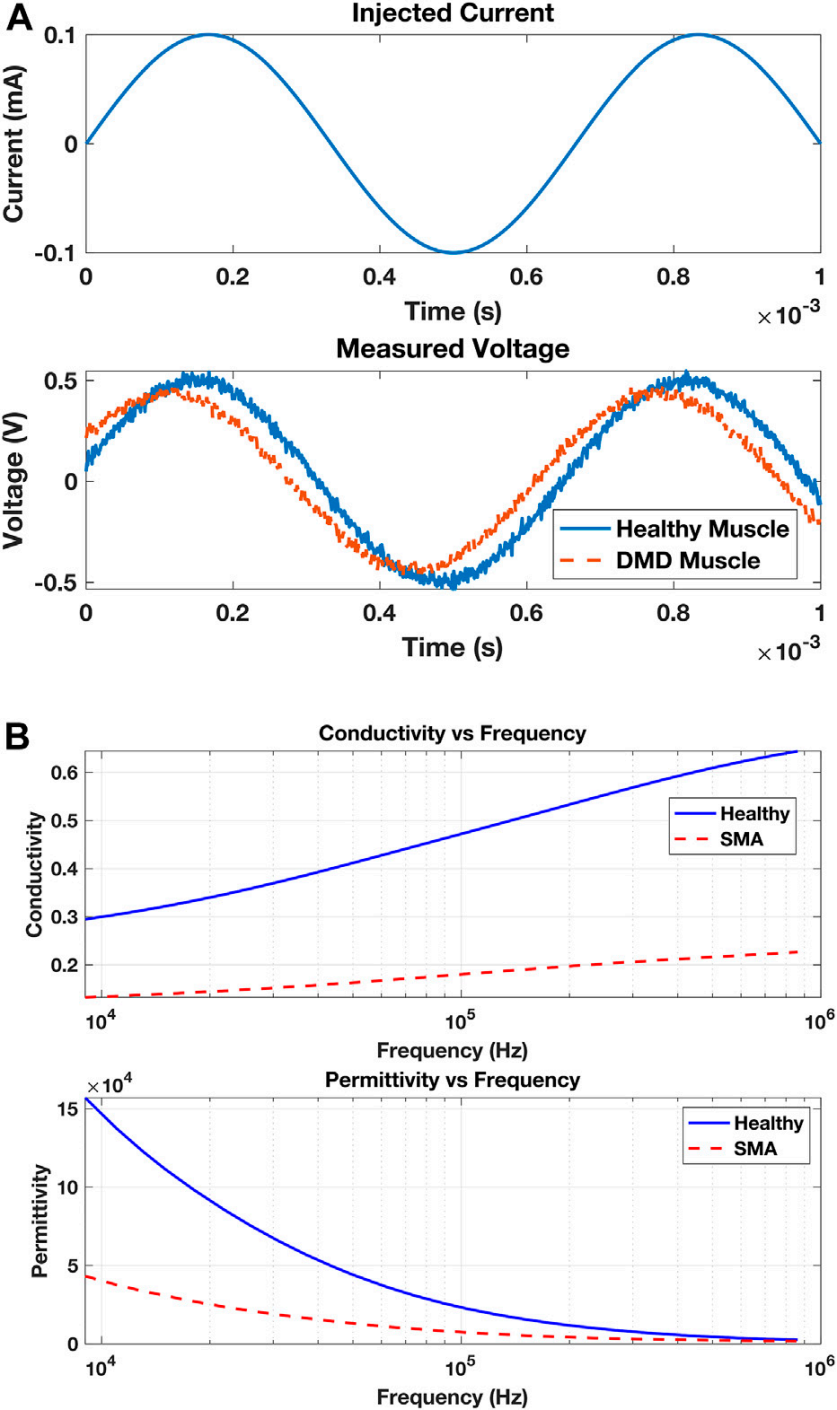
**(A)** A muscle is subjected to an injected electrical current with a specified frequency and amplitude (top). The muscle affects the applied current by varying the amplitude of the measured voltage due to its resistance and slightly adjusting the timing of the voltage due to its capacitance (bottom). The injured muscle (with DMD) shows a phase shift in the measured voltage compared to the healthy muscle. **(B)** Conductivity (top) and permittivity (bottom) variations in the longitudinal muscle of mice that are either healthy (solid blue) or affected by SMA at 40 weeks of age.

EIM measurements play a crucial role in quantifying the pathological status of muscles. As depicted in Figure 3A, the measurement process involves the initiation of a minute current injection of a known frequency (top), with the resulting voltage (bottom) being analyzed to evaluate the muscle’s condition. In comparison to a healthy control, a muscle afflicted with Duchenne muscular dystrophy (DMD) exhibits a discernible phase shift in the measured voltage. Figure 3A illustrates a representative scenario demonstrating the application of EIM in diagnosis; however, it does not present actual experimental data. Further analysis can be conducted by injecting currents at different frequencies and collecting the voltage responses, allowing the construction of several impedance plots. These plots display traces of tissue resistance, reactance, phase angles, and voltage amplitude across the frequency spectrum. In diseased versus normal tissue, changes at the molecular level alter the electrical properties of the tissue, resulting in shifts or shape changes in these traces, which aids in detecting pathological cases. Figure 3B presents data on the longitudinal muscle of mice that are either healthy or affected by spinal muscular atrophy at 40 weeks of age (Sanchez, 2019).

**FIGURE 4:**
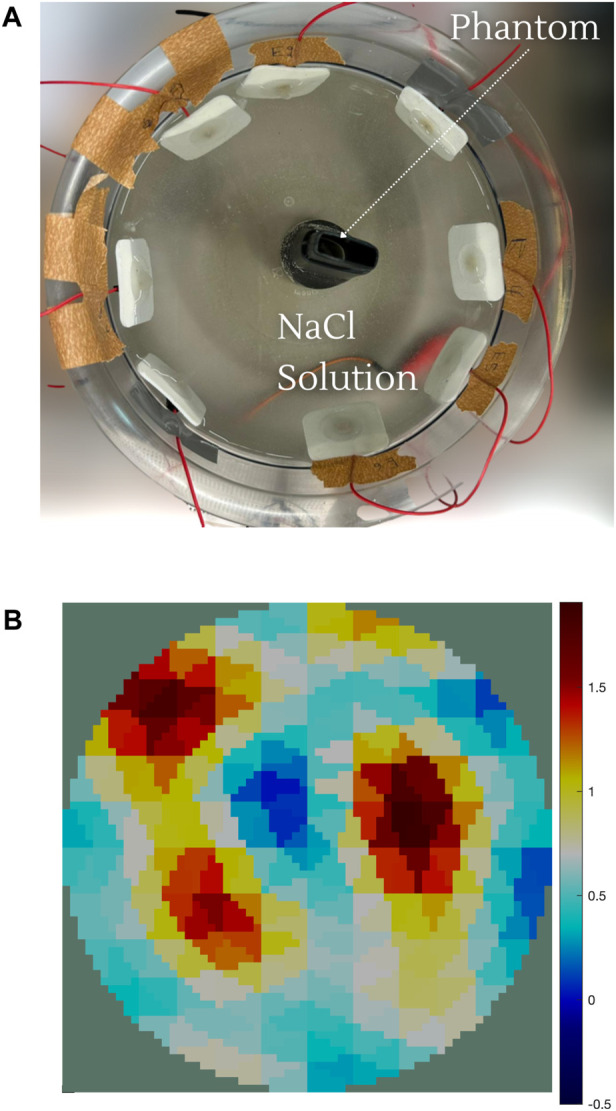
**(A)** EIT experimental setup consists of the basin filled with the saline solution (NaCl and water) with the red dot electrode distribution and the phantom object. **(B)** Image reconstruction of the phantom object. The red areas represent regions with higher concentrations of the NaCl solution, indicating higher conductivity. The blue areas represent the plastic object or regions with lower concentrations of the NaCl solution, indicating lower conductivity.

**FIGURE 5:**
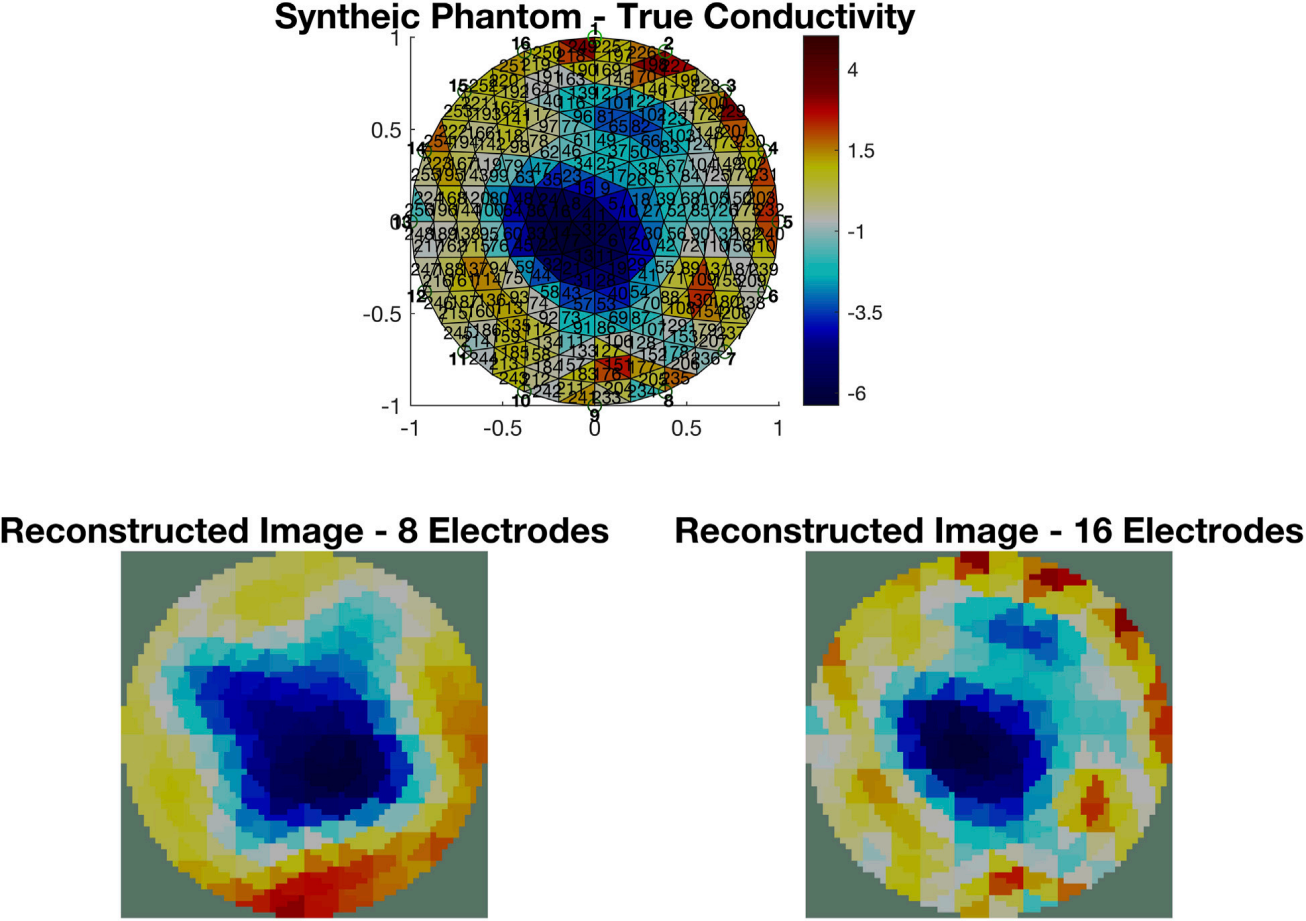
Image reconstruction of a synthetically generated phantom object (top) using EIDORS. The reconstruction using measurements from 8 electrodes (bottom left) has a lower resolution than the one using measurements from 16 electrodes (bottom right).

EIT measurements provide a more descriptive output compared to EIM. A typical EIT experimental setup is shown in Figure 4A, where eight red dot electrodes are used to reconstruct the image of a plastic phantom object shown in the middle. The basin is filled with a saline solution (sodium chloride (NaCl) and water) to ensure conductivity. The 64 adjacent voltage measurements are fed into the Electrical Impedance Tomography and Diffuse Optical Tomography Reconstruction Software (EIDORS) (Adler and Lionheart, 2005), which displays the reconstructed image, as shown in Figure 4B, using the Jacobian reconstruction algorithm. The red areas represent regions with higher concentrations of the NaCl solution, indicating higher conductivity, while the blue areas represent the plastic phantom object or regions with lower concentrations of the NaCl solution, indicating lower conductivity. The resolution of the reconstructed image can be further enhanced by increasing the number of electrodes. The images in Figure 5 show the reconstruction of a synthetically generated phantom object (top) using 8 electrodes (bottom left) and 16 electrodes (bottom right). The increase in the number of electrodes, and in turn the number of measurements, leads to an image reconstruction with improved resolution. The phantom object was synthetically generated using EIDORS, and more details about this software program are covered in Section 8.

## 3 Advancements in the design of EIT and EIM systems

### 3.1 Evolution of EIT devices

The core fundamentals of bioimpedance analysis are the hardware components (on both the macro- and micro-level) in addition to the technique used. Most traditional EIT setups rely on common elements or parts in order to provide the desired outcomes. The procedure for EIT involves passing an electric current through electrodes placed on the target muscle or area and measuring the resulting potential difference. The voltage data are then filtered to extract the amplitude and phase from the injected current (Zhu et al., 2021). Usually, EIT systems use sinusoidal excitation, which allows them to excite the target area and collect data one frequency at a time, using devices such as a field-programmable gate array (FPGA) or digital signal processor (DSP) (Holder, 2005).

Bioimpedance analysis is performed using two devices: electric impedance tomography and electric myography devices. This section covers both modalities. Starting with the EIT devices, the leaders in the development of EIT systems are the Sheffield Group, with their systems ranging from MK1 to MK3.5. Before comparing the different systems, EIT dates back to 1964, when Fatt used two plates of electrodes to squeeze a muscle between them for assessment. The system used consisted of black platinum electrodes, a direct-coupled amplifier, and a network filter to eliminate noise. Injected signals had a frequency range of 1.5 kHz–130 kHz. This system used a Wheatstone bridge for impedance measurement, which was well-known for maintaining high compensation at low levels of frequency (Fatt, 1964). The EIT systems then improved from the release of the Sheffield system to the MK1 device. The latter uses a 64-electrode configuration, which presents a large increase in the number of electrodes compared to the 1964 model without altering the frequency range of 750 Hz and 77 kHz. The model used a PC for data analysis, and the high number of electrodes used facilitated complex brain analysis (Yerworth et al., 2002). Another device that uses a similar range of frequencies and electrode configuration is the OXBACT-5. An interesting aspect of this device is the usage of 16 electrodes for injection and 64 for reading data rather than the 2-by-2 configuration used in the Sheffield MK1 system. Although this device makes use of an FPGA for data processing, it is not bulky and, thus, can be used in clinical applications, which is an improvement over the Sheffield MK1 system (Yue and McLeod, 2008). The release of the MK2 Sheffield system introduced a fixed current injection frequency of 20 kHz and a decrease of 75% in the number of electrodes (4 electrodes). Overall, the system was less bulky than the first generation, much faster, and involved digital data. The designers introduced a data acquisition system with a differential amplifier enhancing the signal-to-noise ratio (SNR) (Oh et al., 2011). A major change in the MK2 systems was the 50% reduction in the current injection amplitude (2.5 mA lower than that of the MK1), which is considered safer. The MK3.5 system, the latest addition to the Sheffield family, switched back to acquiring analog data and passing them through a series of analog-to-digital converters (ADCs) to obtain a digitized input. Data were acquired using eight electrodes. The system is well known for its high SNR and is currently a commercial clinical monitoring device (refer to Table 3) (Wilson et al., 2001). Another system that uses an FPGA as a data processor is the device used by Saulnier et al. (2007), which was developed in 2007; with approximately 72 electrodes and digital data transmission, the device is a well-known modality for breast cancer imaging and diagnostics. This device, which is also referred to as the ACT4 system, has 72 electrodes with an improvement over the Sheffield system, whereby the current injection frequency ranges from 0.3 kHz to 1 MHz instead of fixed frequencies. This system makes use of instrumentation amplifiers to reduce noise and is well known for its flexibility (Liu, 2007). Another device that uses 32 electrodes and a fixed frequency is that proposed by Soleimani (2006), which uses analog-to-digital and digital-to-analog converters for data acquisition and current injection, respectively. The design also integrates instrumentation amplifiers that play a major role in decreasing noise figures, thus obtaining clear images (Soleimani, 2006).

**TABLE 3:** Commercial bioimpedance devices: the table summarizes EIT and EIM commercial devices and their uses along with links to their product pages and manufacturing company.

Product	Company	Description	Uses
Imp SFB7	ImpediMed	Multi-frequency bioimpedance spectroscopy	Clinical and research applications
SKULPT	SKULPT Performance	Multi-frequency EIM Training System	Sports, training, and research
mScan & mView	MYOLEX	EIM	Clinical trials and treatment of neuromuscular diseases
Quantum IV/V/VII	RJL Systems	Full body impedance analyzer	Several clinical and research applications
PulmoVista 500	Dräger	EIT	Pulmonary-related patient care
ENLIGHT 2100	Timpel	EIT	Precision ventilation
LuMon™ System	Sentec	EIT	Clinical pulmonary imaging
MK3.5	MALTRON	EIT	Clinical monitoring

In 2005, the Terason t3000 EIT was developed, introducing lower frequencies, between 20 kHz and 80 kHz, for the injected signals when compared to the previous Sheffield system. The system also used 30 gold-plated electrodes but was hindered by long computational time for image reconstruction (Hueber et al., 2008). Bera and Nagaraju (2012) developed an EIT system with a constant current injecting frequency and 16 electrodes in a circular configuration. The system was the first to be connected to LabVIEW using a PC and MUXs. This system also differed from others since it integrated a voltage-controlled oscillator and a band-pass filter to eliminate noise and amplify and vary the injected signal and its frequency (Bera and Nagaraju, 2012). When developing a wireless EIT system, Huang et al. (2016) attempted to use an improved version of the back-projection algorithm, which required powerful machines to provide a steady current. To reconstruct the images, the researchers used the Maxwell equation since it satisfies the electromagnetic field distribution of biological tissue (impedance algorithm reconstruction). The device was tested on objects inside a phantom, and the results showed that the objects were clearly detected in the reconstructed images (Huang et al., 2016). Lee et al. (2020) also developed another wireless EIT device, which has 4 different ICs, each containing an electrode array of 16 electrodes delivering currents at 1 MHz max with 3 mA-pp. Using bandpass filters in the design improves image accuracy and minimizes noise. Another important EIT device is the KHU device developed by Oh et al. (2007), which uses 32 electrodes (a standard for most devices), but its novelty lies in its ability to inject currents at frequencies in the range of 10–500 kHz throughout the electrodes. The device was further enhanced into the KHU-Mark2 system, which had an acquisition speed of of almost 100 scans/s, faster than the KHU-Mark1 model. The enhanced performance is mostly due to the addition of independent current sources and voltmeters (Oh et al., 2011). The final model of this series was the KHU-Mark2.5, which included a module for self-calibration, allowing the reconstruction of images with better clarity than previous devices (Wi et al., 2013). One of the newer models of EIT systems is the KIT4 model, which has a different electrode configuration system; it consists of separate and independent recording and injection modules. The recording modules can acquire 80 signals simultaneously, while the injection module has 16 channels, which allow for a 2D system highly sensitive to conductivity (Hauptmann et al., 2017).

Although EIT is not commonly used for brain imaging, the device developed by Shi et al. (2018) mainly focused on acquiring data through brain imaging. The device had common electrode numbering and a circular configuration. Data transfer and acquisition for both recording and injection used ADCs and digital-to-analog converters (DACs), respectively. The device also had a programmable current source so that the data acquisition system could compensate for the shunt effect and excited current. The current source could provide a current with an SNR of 89 dB (Shi et al., 2018). Most EIT systems make use of a PC for data processing, but the device developed by Zamora-Arellano et al. (2020) made use of an Arduino and a raspberry pi for current injection control and signal processing, and no significant differences were noticed between their work and related works in terms of speed and resolution. Another device that uses an Arduino for data acquisition was developed by Zhu et al. (2021) and used 32-to-1 analog MUXs and a current injection rate of 500 kHz, which significantly increased the acquisition speed of the device.

### 3.2 Evolution of EIM devices

After covering all advancements regarding EIT, this section will now elaborate on the advancements in EIM. As mentioned earlier, a notable difference between EIM and EIT devices is the decreased number of electrodes. EIM devices are commonly used to diagnose neuromuscular diseases. For example, the study conducted by Tarulli et al. (2006) to investigate neuromuscular diseases involved an EIM device consisting of 4 Ag/AgCl electrodes in an inject/receive configuration, connected to a PC for processing. The device uses a lock-in amplifier that can provide signals ranging between 2 kHz and 2 MHz (Tarulli et al., 2006). Although typical EIM systems sample at a frequency of 50 kHz, Shiffman et al. (2008) developed an EIM system capable of reaching up to 2 MHz, which is useful in distinguishing healthy muscles from diseased muscles. This creates high common-mode noise and thus requires the use of ADC with high precision to detect small range differences. The device also uses distant electrodes for injecting current (Shiffman et al., 2008), a common EIM injection method mentioned earlier (Rutkove et al., 2005). To study the bioimpedance of thoracic injury, Buendia et al. (2017) used a bioimpedance device that makes use of four gel electrodes and a single injection frequency of 50 kHz. This device offers quick data configuration but does not provide a frequency range.

**FIGURE 6:**
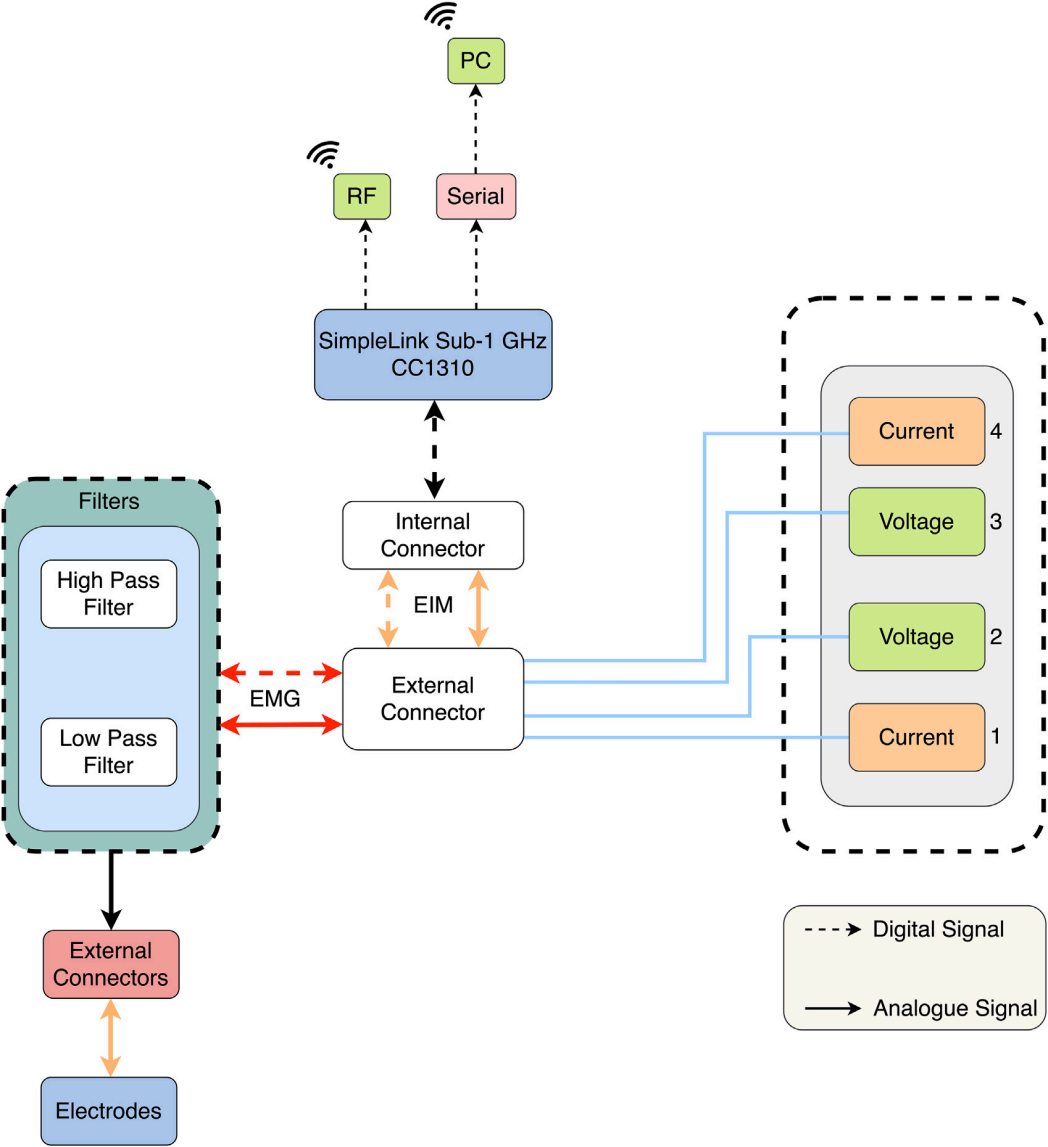
Block diagram of the EIM-driven EMG system (Ngo et al., 2022).

It is possible to combine EIM with other technologies and observe multiple physiological parameters. For example, Ngo et al. (2022) used EIM as another source of data collection for EMG devices. The device proposed offered 4 channels, each composed of 16 wet electrodes, with two outer electrodes used to inject the EIM current data (Figure 6). The inner electrodes were used for measurements for both devices, EMG and EIM. In addition, for data processing, the signal was sent to a PC with Intel processing, which is a commonality between all EIM devices. Furthermore, in this configuration, the EMG signal was post-processed using a second-order band-pass filter, while the EIM was pre-filtered using a low-pass filter (Ngo et al., 2022). Another example of multi-purpose devices is that of Hornero et al. (2013). They used an EIM device with four dry-contact copper foil electrodes, with an inject/read configuration similar to previously mentioned devices. The device was developed to allow the observation of amputees. Using the 50 kHz injection, this system is capable of monitoring both heart and breath activity through electrical impedance plethysmography (IPG) measurements and muscle activity through EIM measurements, with a single long-term use system (Hornero et al., 2013).

### 3.3 Design components of EIT and EIM devices

**FIGURE 7:**
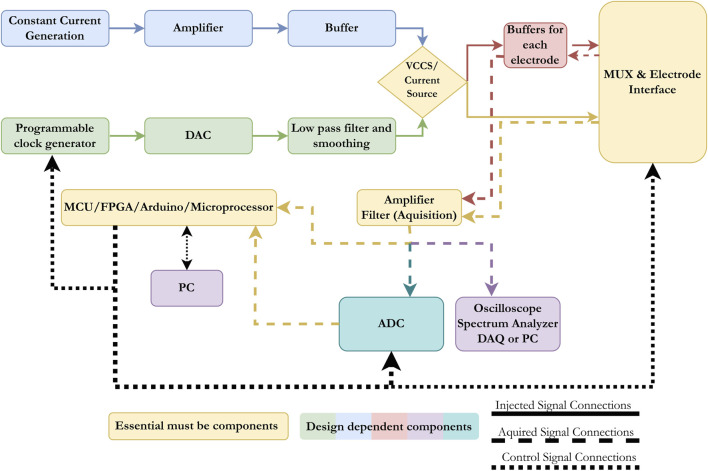
Block diagram presenting the main components in any electrical impedance devices (EIT or EIM). The basic components are a current source, an electrode interface unit, an acquisition unit, and a control/processing unit.

After reviewing the most recent electrical impedance systems developed, we attempt to summarize the main building blocks in designing any EIM or EIT system. The basic components of any system are as follows: (1) the injection unit that delivers current to the target organs, (2) the interface unit that transfers the injected current to the target and delivers recorded signals to the processing unit, (3) the recording and processing unit, which amplifies, filters, and enhances recorded signals, and (4) the main control unit, which synchronizes injection and recording, transfers data to PC or post-processes them, and, in some cases, controls the injected current. These units are shown in yellow in Figure 7. Signal injection can be carried out through a current source or a VCCS. In the first method, the analog signal is generated by a constant current generator, passed through an amplifier and buffer, and sent to the injection electrodes either directly or through buffers for each electrode depending on the design of the system, the noise levels, and the used frequency. As for the VCCS, a digital signal is generated by a programmable clock generator, converted to an analog signal, filtered, smoothed, and then injected either directly to the electrodes or through another set of buffers. The programmable clock generator is controlled through an MCU, FPGA, Arduino, or microprocessor, depending on the choice of the designer. In systems that use the same electrodes for injection and recording, a switching component is required. Most systems rely on MUXs to transition between injection and recording or, in some cases, simple relays are used. The switching event is also controlled by the processing units mentioned earlier (MCU, FPGA, Arduino, or microprocessor). The recorded signals are often low in voltage and noisy in nature, and therefore, most systems use amplifiers and filters to enhance the quality of the obtained signal before any analysis is done. The signal is either transferred to an oscilloscope or spectrum analyzer or converted to a digital signal, then to a processing unit or PC, or directly transferred to a processing unit. The factors that affect the selection are the quality of the obtained signal, the frequency range, and whether the end purpose is impedance analysis or image reconstruction. It is important to mention that a wide range of filtering techniques can be used for both the injected and recorded signals. In the literature, the most commonly used methods rely on instrumentation amplifiers due to the high common-mode rejection ratios (CMRRs) they produce and their high accuracy, even with long-term applications.

### 3.4 Commercial devices

Currently, there are many commercially available electrical impedance devices. Table 3 summarizes commercial systems available with links to the devices and their producing companies. It should be noted that many of these companies have other impedance devices that are either outdated or no longer produced but have appeared previously in the literature, such as the MK devices previous to MK3.5.

## 4 Reconstruction and signal processing techniques

### 4.1 EIT-based algorithms

**FIGURE 8:**
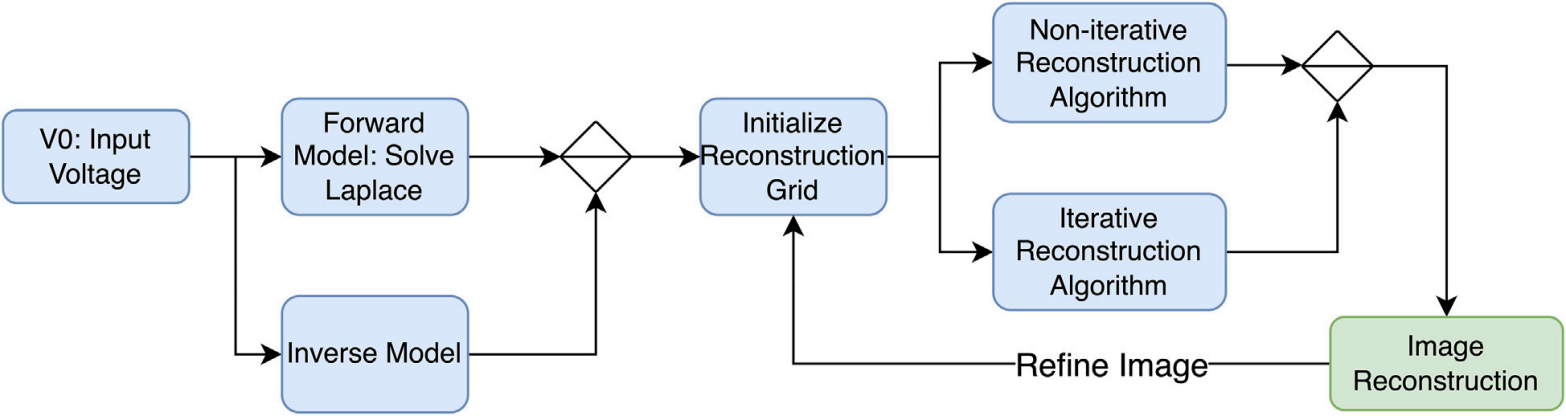
Image reconstruction process—block diagram.

Generally, when using EIT as a medical technique for producing images, two important factors determine the quality of the obtained image. First, the number of electrode measurements taken, which was previously discussed, and the type of reconstruction algorithm used. This section will cover all types of algorithms for reconstruction purposes and advancements and previously used methods in the field of EIT. To start, the main point to know is the forward problem. The forward problem can be defined as the process of obtaining boundary voltages for current patterns and resistance distributions. Apart from the forward problem, there is the inverse problem that attempts to find the admittance distributions from a finite number of boundary voltage measurements. A representation of the process of the reconstruction of an image using electric impedance tomography is shown in Figure 8.

**FIGURE 9:**
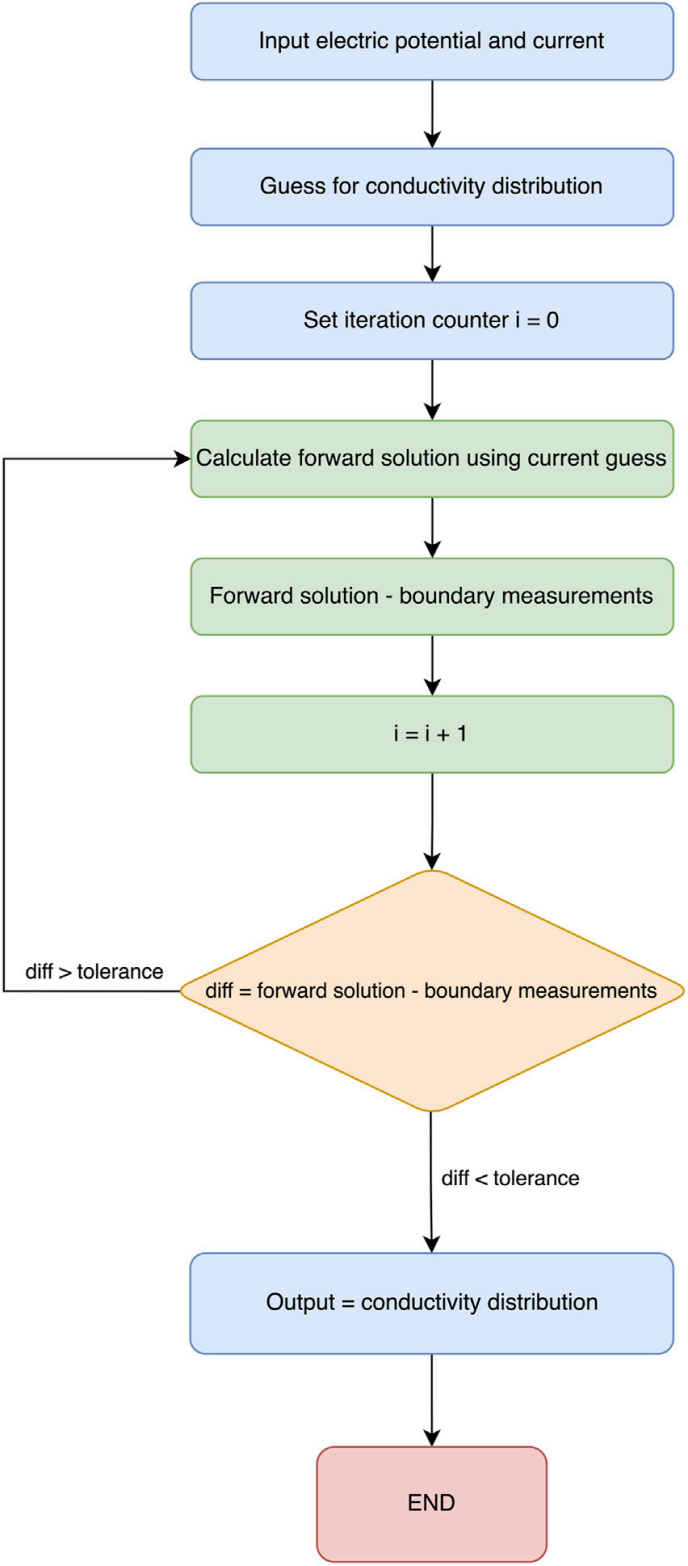
Newton–Raphson flow diagram.

EIT reconstruction algorithms can be divided into two sections: (1) iterative, which are optimization-based methods that iteratively refine an initial estimate of the conductivity distribution until it converges to a solution that best fits the measured data, and (2) non-iterative, which are also known as direct or algebraic methods that aim to directly solve the EIT reconstruction problem in a single step without iterative refinement. While both algorithms involve the inverse problem, iterative solvers require repeated calls to the forward solution, whereas non-iterative solvers rely on pre-computed Jacobians and/or forward solutions. One of the most popular algorithms used to reconstruct EIT images is the Newton–Raphson algorithm. The latter generates a Jacobian inverse problem, which can be solved using Tikhonov regularization (Nofrianto et al., 2021). In his study, Abdullah (1999) attempts to solve both problems using the modified Newton–Raphson (MNR) algorithm. Initially, this algorithm iterates to a final solution by updating an initially guessed admittance with respect to an error. The algorithm repeats this process until an acceptable solution of the admittance of distribution is achieved, and tests on computer-generated data patterns show that the algorithm takes four iterations to converge. Figure 9 shows a flow chart explaining the process. The results show that it is possible to solve the inverse problem using MNR. One limitation to this algorithm is the cost of building the data acquisition system to be used in MNR compared to other techniques (Abdullah, 1999).

Somersalo et al. (1992) employed a distinct algorithm in their study, focusing on the utilization of a complete electrode model (CEM). Within their investigation, they scrutinized various electrode models, with particular emphasis on the complete electrode model. They delved into the imperative task of identifying the optimal electrical property distribution within the object and aligning it with the measured current and voltage data. To achieve this, they utilized the finite element method (FEM) to obtain the numerical solution for the CEM model, while concurrently addressing the known electrode geometry and properties (Somersalo et al., 1992). Yong et al. (2021) evaluated the use of the Graz consensus reconstruction algorithm for EIT (GREIT), a linear reconstruction algorithm, for lung EIT images. One issue in lung image reconstruction is the low spatial resolution. To solve the issue, researchers incorporated the FEM with GREIT and classified the obtained image using fuzzy logic based on GREIT test parameters: amplitude response (AR), position error (PE), ringing (RNG), resolution (RES), and shape deformation (SD). The results were constant and uniform; thus, the images obtained were much clearer (Yong et al., 2021). Sharma et al. (2020) conducted a study to determine whether using a gravitational search algorithm (GSA) in EIT image reconstruction can help in the determination of bladder size. The results showed that the bladder size and shape were recovered with good accuracy; thus, their proposed theory proved to perform well (Sharma et al., 2020). Li et al. (2014) used the FEM with an alternative mesh refinement algorithm, which resulted in a better image spatial resolution compared to the images generated from the Gauss–Newton method.

An alternative way of solving the EIT forward problem is to minimize the relative error between surface potentials. An algorithm that accomplishes this goal is the genetic algorithm (GA) and Fish school search (FSS) algorithm. Using these algorithms, the images were consistent, but GA gave a better image resolution (dos Santos et al., 2018; Martin and Choi, 2015). Martin and Choi (2015) showed that using particle swarm optimization and an artificial neural network, images can be reconstructed faster with higher visual fidelity than when using the Gauss–Newton method.

Initially, algorithms used for thorax reconstruction assumed that the electrodes were placed on a circular surface, while new algorithms utilize information about the shape of the thorax. In a study conducted by Tarabi et al. (2021) to measure solid volumetric scales, researchers used the FEM mesh algorithm to solve the forward problem in EIT and then applied a modified Newton’s One-Step Error Reconstructor (NOSER) style regulation to recover a change in conductivity in the measured spaces. They found that this approach reduces processing times when compared with Tikhonov algorithm and GREIT (Tarabi et al., 2021). Using the damped least-squares method with Cheney’s NOSER algorithm, Shi et al. (2018) improved the stability of the reconstructed brain images. Yao et al. (2019) developed a wearable EIT system (wEIT) and investigated a new way of solving the forward problem using sampling. They found that this algorithm overcomes the different drawbacks of the iterative algorithms by obtaining the minimum angle between the input vector and solution vector. Daneshmand and Jafari (2013) investigated the 2D method and proposed a 3D boundary element–finite element (BE–FE) solution for the forward problem. To validate their method, researchers attempted to solve a homogeneous test problem. The results showed good agreement between the FE and BE and FE–BE methods and the exact solution. Thus, they determined that this method can use the advantages of both BE and FE methods and reduce the dependency of the solution on the size of the reconstructed shape (Daneshmand and Jafari, 2013).

A study conducted by Borsic and Adler (2012) on prior dual interior-point methods (PDIPMs) for efficiently using the total variation (TV) function in EIT found that TV regularization algorithms produced sharper images of medical data than those with quadratic regularization algorithms. Jin et al. (2012) developed a sparse reconstruction algorithm based on the Tikhonov function. After testing, the results showed that the proposed technique could yield acceptable reconstruction results compared with conventional approaches. Zhou et al. (2015) compared the performance of different TV algorithms, such as the PDIPM, linearized alternating direction of method of multipliers (LADMM), and split Bregman (SB). Their results showed the fastest calculation speed but the worst resolution. PDIPM showed the sharpest change in conductivity reconstruction. SB had a faster convergence rate than PDIPM and the least imaging errors (Zhou et al., 2015).

After discussing the iterative algorithms, this section highlights the ones that are most widely used. A popular algorithm in the literature is the back-projection Sheffield algorithm, which generates an EIT sensitivity region and determines the required information from the image (Putensen et al., 2019). The inverse reconstruction using a single set of voltage measurements is a process that is very sensitive to deviations. To avoid the previously mentioned problem, Wagner et al. (2022) applied the FEM method and obtained images of the spatial strain of large areas. When creating a wireless EIT system, Huang et al. (2016) employed an enhanced back-projection algorithm, necessitating robust hardware to maintain a consistent current flow. For image reconstruction, they utilized the Maxwell equation, which accurately represents the electromagnetic properties of biological tissues (impedance algorithm reconstruction). Testing the device on items within a phantom demonstrated that the reconstructed images distinctly identified the objects (Huang et al., 2016). EIT has low image resolution compared to other imaging techniques, such as CT. To address this issue, Gomes et al. (2020) suggested a different reconstruction technique that involved the use of autoencoders to replicate input electric potentials and deep neural networks with the extreme learning machine (ELM) to apply the back-projection algorithm. Furthermore, when testing an EIT system that decomposes body tissue into resistive (real) and conductive (imaginary) parts, Sapuan et al. (2020) used the linear back-projection algorithm. The reconstruction resulted in a functional image. Functional images are split into two images: the impedance image and the resistive image; thus, the development of the EIT system was successful (Sapuan et al., 2020).

The most common reconstruction technique in EIT is difference imaging. Some common algorithms for reconstruction are the shape constraint imposed method and the 1D D-bar method, but these methods lead to systematic errors and uncertainties. Liu et al. (2020) proposed reconstructing the images based on the shape reconstruction problem and solved it via geometrical methodology. After testing their algorithm on phantom and pig data and comparing them with the regular linear approach, researchers found that their approach produced greatly improved images compared to the conventional linear approach (Liu et al., 2020). Additionally, Yuan (2020) tested the use of an improved FEM algorithm called the filtered back-projection algorithm. This model was constructed using LabVIEW. This optical projection tomography (OPT) reconstruction provides structural information such as size and shape of the image (Yuan, 2020). To obtain a clear scan of the chest using EIT, Bachmann et al. (2018) used a new technique that involved reconstruction using FEM mesh analysis and then projecting the images in a 32 × 32 array of pixels; the image obtained showed the healthy and collapsed lungs accurately, and researchers were able to assess acute respiratory distress syndrome (ARDS) in patients. Another algorithm is the 2D D-bar algorithm. Hamilton and Hauptmann (2018) showed that the 2D D-bar method for EIT was more reliable for solving the inverse problem than the 1D D-bar method, but the reconstructed image suffered from blurring. Adding a convolutional neural network (CNN) helps in better image recovery with minimal added time to the post-processing of the image (Hamilton and Hauptmann, 2018). It is worth noting that non-iterative algorithms are generally faster than iterative algorithms but may not produce as accurate results as iterative algorithms.

Non-regular alternatives for solving the forward and inverse problems can include machine learning and different software tools. Kłosowski and Rymarczyk (2017) proposed using a neural network that is trained using a Bayesian model; this method does not involve any preliminary assumptions. The method considers each image as a set of pixels with specific conductivity to reconstruct, which allows for fast image reconstruction (Kłosowski and Rymarczyk, 2017). Additionally, Fernández-Fuentes et al. (2018) developed a machine learning model to solve the inverse problem of EIT. The results from the proposed method were analyzed quantitatively and qualitatively and compared with PDIPM Gauss–Newton algorithm. This method resulted in a good kappa index and an accuracy of 97.57% and 94.6%, respectively (Fernández-Fuentes et al., 2018).

Another method of solving the inverse and forward problem for image reconstruction is using EIDORS software. This software program was specifically designed to reconstruct images back from EIT (Adler and Lionheart, 2006). The main focus was to investigate the use of internal electrodes to generate 3D images from EIT. In particular, they investigated the use of a 3D GREIT reconstruction algorithm using EIDORS software. Their results showed that the obtained reconstructed images using the internal electrodes and 3D reconstruction algorithm resembled the objects used more than the obtained images when 2D EIT was used (Stowe and Adler, 2020). Further studies were conducted by Bera and Nagaraju (2012) to identify chicken tissue in a phantom using EIT. To solve the forward problem, researchers used a modified mesh of the FEM EIDORS algorithm to suit the geometry of the phantom. They were successfully able to reconstruct the resistivity of the chicken tissue phantom (Bera and Nagaraju, 2012). Additionally, Soleimani (2006) made use of MATLAB to design a 2D EIT system for image reconstruction. This program was able to generate meshes for the FEM model. The Raphson–Newton method was used for image reconstruction. The system was tested with different approaches, such as simulation and real-time measurements; when tested with a phantom, the system generated satisfactory results as the reconstructed images enabled the detection of the object inside the saline solution. A limitation to this study was the number of projections used in the back-projection algorithm, which limited the quality of the image (Soleimani, 2006).

### 4.2 EIM-based algorithms

EIM is usually performed using four electrodes to maintain the signal quality. The response of the tissues to an electric stimulus is recorded by the electrodes. The resulting signal’s impedance Z and phase angle θ are processed using the ratio and normalization of the voltage and current amplitudes and phase delays. To establish a relationship between EIM, myofiber, and physiology simulations, a bio-physiological model that explains the EIM data is used (Sanchez and Rutkove, 2017a). A representation could be the Cole model, which divides the frequency dependency of EIM into four parameters (Levenberg, 1944; Marquardt, 1963). EIM parameters usually comprise R , X , PA , and Z . X consists of a combination of two forms, the capacitive reactance $\left(X_{C}\right)$ and inductive reactance $\left(X_{L}\right)$ . PA is calculated using standard trigonometric equations. Z is the complex impedance obtained by the resistance as the real part and the capacitance as the imaginary part. Wang H. et al. (2021) made use of the ImpediMed SFB7 multi-frequency analysis system to process the obtained parameters from the lumbar paraspinal muscles (LPMs) of patients suffering from chronic lower back pain (CLBP). The results showed that the R and Z parameters were elevated in patients with CLBP, while X was similar to the control sample and PA was decreased. A major limitation to this study was the small sample size as measurements were only taken from young adults (Wang H. et al., 2021). Semple et al. (2020) conducted a study to assess muscle changes in animals exposed to hindlimb suspension using a pelvic harness (HLS) and a partial weight-bearing (PWB) model that mimics partial gravity, including lunar and Martian gravities. They performed *in vivo* impedance measurements on rats and obtained the phase (LP) , reactance (LX) , and resistance (LR) as the output. Multi-frequency analysis was done on the previously mentioned parameters in a range of 100−500kHz . After the analysis, they concluded that EIM can be used to detect muscle-bearing alterations in rats (Semple et al., 2020).

Ngo et al. (2022) developed a wearable multi-frequency device to measure muscle activity. This device combines EMG with EIM, where the system performs measurements at a range of 2 and 200 kHz; thus, the system would derive the bioimpedance from the signal— $Z_{\mathrm{EIM}}$ ( Ω ). This can be used to investigate human muscle contraction (Ngo et al., 2022). In this system, the EIM module must detect the bioimpedance between 20 Ω and 200 Ω with an injection frequency of 140 Hz. To test the device, they scanned a human leg test subject. The EIM module’s accuracy was measured alone and was found to have an error of 0.8%. Thus, they concluded that the EIM module can measure bioimpedance with an error of less than 5% at 140 samples per second (SPS); thus, it can replace SFB7 (Ngo et al., 2022). EIM techniques expanded to involve multiple frequencies rather than the 50 kHz frequency (Aaron and Shiffman, 2000). Shiffman et al. (2008) designed an EIM device that can be used at a frequency of 2 MHz. The device contains a voltage divider and rotates the effective source away from the real axis into the complex plane. To study the body impedance of the thigh of a female patient, Cole and Cole (1941) made use of the Nyquist plot of X(f) and R(f) . Additionally, Kramers and Kronig (K–K) developed the K–K relations that can rule out the discrepancy between reactance and ‘3-element behavior’. Additionally, it states that the difference between the initial reactance and the infinite one is represented as the inverse of the frequency (Kronig, 1926; Kushner, 1992).

### 4.3 Combination of EIT with other modalities

In the realm of medical imaging, EIT has paved the way for a fascinating array of hybrid modalities, such as magnetic resonance electrical impedance tomography (MREIT) and acousto-electric impedance tomography (aEIT), each harnessing the power of diverse physical principles to uncover a deeper understanding of the body’s internal structures and functions.

Using MREIT, current injected into an object results in a magnetic flux. To reconstruct the image of the object, Hamamura and Muftuler (2008) used the sensitivity matrix method (SMM), which discretizes the object domain into a mesh of triangular elements. The results showed that the MREIT was able to map the general variation in the magnetic flux density. Martins and Tsuzuki (2015) proposed a multi-objective optimization algorithm based on the simulated annealing method for EIT image reconstruction. To evaluate the proposed algorithm, researchers performed the reconstruction experiment on a phantom filled with three cucumbers, they compared the results of their algorithm to the archived multi-objective simulated annealing, and found coherence between the two (Martins and Tsuzuki, 2015). Furthermore, Kim et al. (2007) used the harmonic Bz (z-component of the magnetic field) algorithm to obtain postmortem canine brain images via MREIT, which revealed a distinct contrast between the conductivity of white and gray matter. They also conducted *in vivo* imaging experiments on canine brains with and without regional brain ischemia and found that the ischemia affected conductivity measurements (Lee et al., 2017). In addition, researchers conducted an MREIT experiment on a human brain, where they observed excessive noise in the outer layer of the cranium (Kim et al., 2007). Moving on to aEIT, it combines ultrasound and electrical impedance measurements to create images based on the interaction of acoustic waves with tissue conductivity changes. This approach allows for the visualization of electrical conductivity variations within biological tissues. A famous type of aEIT is the Lorentz force EIT, which uses granular spectral patterns that appear in ultrasound imaging to distinguish and identify pathologies. A study done by Grasland-Mongrain et al. (2015) consisted of ultrasound speckle and ultrasound imaging on a bovine rib muscle; their results showed a speckle pattern in the first technique mentioned, which revealed two types of information, acoustic and electric homogeneities. The speckle pattern observed in both techniques used showed similarity, thus allowing them to conclude that aEIT using Lorentz force can be used to study electric inhomogeneity structures (Grasland-Mongrain et al., 2015). Additionally, Haider et al. (2008) investigated the use of magneto-acousto electric impedance tomography (MAET), and their study showed high spatial resolution images.

## 5 Electrical impedance tomography

The early diagnosis of diseases is critical to achieving high recovery rates for many illnesses. Imaging modalities such as X-rays, ultrasound (US), magnetic resonance (MRI), and nuclear-computed tomography (CT) are commonly used diagnostic tools. Despite their success, many of these tools are invasive, have low resolution that is affected by the operator’s skills, and put the patient at risk of exposure to radiation, electromagnetic waves, or contrast agents causing allergic reactions. Additionally, these modalities are often bulky, require long image processing time, and some cannot be used in the presence of pacemakers or metallic bone replacements. EIT is a rising, non-invasive, portable imaging modality that provides insightful information about the target organ based on the collected bio-impedance data. This section will include studies using EIT to diagnose diseases and monitor specific organs.

### 5.1 EIT for detecting lung diseases

In recent years, there have been significant advancements in the imaging assessment of patients with different lung diseases. EIT makes use of electric current to evaluate the distribution of current conductivity in the thoracic activity (Tomicic and Cornejo, 2019). To monitor the lungs using EIT, multiple measurements are taken from a high-frequency (50–80 kHz) and low-intensity (5–10 mA) alternating current that is distributed among an abundance of electrodes placed on the thoracic cage of the patient. Most impedance changes occur in the $5^{th}$ to $6^{th}$ intercostal spaces at the parasternal line. This allows for the detection of different respiratory diseases, such as acute respiratory distress syndrome. According to Holder (2005), an increase in EIT use has been detected in patients with chronic and spontaneous breathing difficulties, most of which are done in small clinics, showing a promising future for EIT in lung disease detection. The need for continuous bedside assessment of the exact pulmonary deficiencies in intensive care patients led Frerichs et al. (1998) to investigate whether EIT could provide the previously mentioned requirement. As a result, they concluded that functional EIT has a future in lung applications since they were able to correctly localize regional ventilation distribution. When EIT monitoring was used on a patient admitted to the ICU, researchers obtained an asymmetrical image of the lungs with almost no sign of ventilation-related impedance changes; thus, they determined that the accuracy of EIT is compromised in an ICU or operating room (Frerichs et al., 1998). The increased frequency of respiratory failures among intensive care patients poses the need for different monitoring tools, such as EIT. In their study, Maciejewski et al. (2021) assessed lung overdistension (OD) and collapse (CL) using incremental/decremental positive end-expiratory pressure (PEEP) trials in which they progressively increased PEEP values up to 20 cm H_2_O and then decreased them stepwise by 5 cm H_2_O. An EIT device was used for monitoring and determining where OD and CL occur simultaneously (OD/CL compromise), which occurs at almost 11.6 ${\text{cmH}}_{2}$ O of PEEP. Thus, they concluded that EIT helps assess the effectiveness and safety of mechanical ventilation in acute respiratory failure (Maciejewski et al., 2021). Although computed tomography (CT) scans are commonly used for classifying ARDS, EIT has emerged as a new technology that is capable of optimizing pulmonary monitoring, especially in patients with ARDS (Bachmann et al., 2018). A study conducted by Gibot et al. (2021) on avoiding alveolar collapse while personalizing PEEP for ARDS and Sars-CoV-2 (COVID-19) patients showed a uniform ventilation distribution, minimizing silent spaces and lowering lung collapse and distension. The PEEP values differed from conventional ones obtained from higher PEEP/ ${FiO}_{2}$ and PL/ ${FiO}_{2}$ but showed no significant difference in respiratory mechanics compared to lower PEEP/ ${FiO}_{2}$ (Gibot et al., 2021). Although the study showed that EIT allows for a personalized PEEP titration to decrease pulmonary spaces, it still had major limitations since the sample size was too small to generalize and only included patients suffering from COVID-19; thus, the study lacked control subjects (Gibot et al., 2021). Bikker et al. (2010) found that EIT-derived parameters, such as $\Delta Z_{\mathrm{global}}$ and $\Delta Z_{\mathrm{regional}}$ , which represent the tidal oscillation in the global plethysmogram caused by each respiratory cycle, and P/V curves, are helpful in reducing alveolar overdistension using different maneuvers such as PEEP-trials. It has been shown that EIT can be used to assess the usefulness of prone positioning in ARDS patients (Riera et al., 2013). Furthermore, Scaramuzzo et al. (2020) conducted a study to identify whether prone positioning helps in reducing mortality rates in acute respiratory distress syndrome. Researchers tested the effects of prone position on perfusion using EIT. The results showed a marginal effect on perfusion redistribution in the lungs. The study presented a few limitations as the EIT data were incomplete; thus, researchers were unable to obtain definitive conclusions from the study (Scaramuzzo et al., 2020). Tomasino et al. (2020) obtained conflicting results when detecting ARDS in healthy patients with a slight increase in ventilation in the dorsal area. In another study conducted by Scaramuzzo et al. (2021), EIT was used to detect transpulmonary driving pressure ( ${\text{DP}}_{L}$ ) and, in turn, assess lung elastance (EL) in patients affected by ARDS. They were able to successfully predict ${\text{DP}}_{L}$ and EL using EIT with 40% accuracy. Additionally, integrating EIT generated accurate results with the esophageal pressure (Peso) monitoring technique proved that researchers measured the previously mentioned parameters using EIT and Peso on a patient undergoing PEEP titration. The EIT and Peso-derived measures showed a bias of $-1.4e^{-007}$ and a limit of agreement (LoA) of −2.4 to 2.4 ${\text{cmH}}_{2}$ O. In other words, if we were to measure a patient’s respiratory parameters using both EIT and Peso-derived methods, we could expect the two measurements to be very close to each other on average, but there could be individual cases where the difference between the two methods is as large as 2.4 ${\text{cmH}}_{2}$ O (Scaramuzzo et al., 2021). Although EIT can be considered an accurate tool for ARDS bedside monitoring, this study excluded patients suffering from COVID-19. Thus, the previously mentioned technique cannot be applied to COVID-19 patients with ARDS (Scaramuzzo et al., 2021). Using EIT imaging, total liquid ventilation (TLV) showed promising results in replacing regular mechanical ventilation and avoiding lung injury. The main limitation to this study was the use of saline injection, which affected the electrical properties of the lungs (Sage et al., 2018). Yuan et al. (2021) examined the effect of position change from bedside to a wheelchair in patients suffering from respiratory failure. EIT proved to be an efficient tool in monitoring the regional ventilation distribution changes, where it accurately detected the increase in ventilation in the dorsal region of the lungs during the change in position from the bedside to wheelchair (Yuan et al., 2021). Apart from ARDS detection, EIT continuous monitoring allows for the detection of lung perfusion (PE). Wang X. et al. (2021) showed that EIT could detect prominent ventilation defects. Additionally, EIT data matched the data provided by a CT pulmonary angiography. Xu et al. (2021) elaborated on the methods for assessing PE using EIT. The authors explained the pulsatility method, which is widely used in the literature, and the conductivity contrast saline (bolus) method and its progress in the field. They have found that the bolus method showed strong diagnostic efficiency as it could detect imbalanced ventilation in patients affected by COVID-19. The limitations to this study include (1) the lack of details to provide a vertical location of perfusion and (2) the lack of optimization in the reconstruction algorithms (Xu et al., 2021). Patients with chronic obstructive lung disease (COPD) develop ventilation inhomogeneity, which is usually detectable using a CT scan. A study done by Frerichs et al. (2021) on 52 patients, 38 with COPD and 14 healthy test subjects, using EIT to detect ventilation inhomogeneity showed that EIT can determine the pathologically increased ventilation heterogeneity in COPD. In addition, it was proved that EIT can detect the forced full expiration maneuver, the forced full inspiration maneuver, and quiet tidal breathing (Frerichs et al., 2021). Bluth et al. (2019) investigated the replacement of PET scans with EIT monitoring in patients suffering from respiratory disorders to track lung perfusion. Saline solutions were used for perfusion, simulating real clinical values. It was shown that EIT overestimated the perfusion in some regions and underestimated it in dependent regions (Bluth et al., 2019). Sun et al. (2020) showed that EIT is a reliable method to assess de-recruitment in lung volume $V_{\mathrm{DER}}$ using the multiple pressure-impedance curves derived from monitoring data. A study by Shono et al. (2017) on the importance of EIT monitoring in preventing ventilator-induced lung injury (VILI) showed that EIT data allowed doctors to administer muscle relaxation to a patient exerting great inspiratory effort during mechanical ventilation. Costa et al. (2008) proved that EIT can reliably detect the development of pneumothorax at the bedside in real-time. EIT can detect pneumothorax within three ventilator cycles with 100% sensitivity. Mechanical ventilation due to the use of positive pressures may itself lead to pneumothorax (Morais et al., 2017). Although EIT still requires more validation to be fully supported by researchers as an alternative to other imaging techniques, the literature clearly shows its potential to become a standard monitoring tool for personalized ventilatory care and patient safety (Maciejewski et al., 2021).

### 5.2 EIT for cancer diagnosis

Studies have shown that the electrical properties of malignant cancerous tissues differ from those of healthy tissues. Thus, bioimpedance analysis is a suitable candidate for cancer detection, especially in breasts or the prostate (Wu et al., 2021). Cancer imaging with bioimpedance analysis devices, specifically EIT, has mainly focused on static imaging, which requires stricter requirements on the data acquisition system in terms of error and channel-to-channel accuracy (Holder, 2005). In the case of prostate cancer, the most common detection method is transrectal ultrasound (TRUS), yet this method has limited accuracy (Wan et al., 2013). To tackle the previously mentioned deficiency, Borsic et al. (2010) developed an ultrasound-coupled transrectal EIT (TREIT). Using the TREIT method, researchers were able to identify the difference in electrical properties between benign and malignant tissues. They state that TREIT may be an effective tool for identifying large tumors in the coarse mesh. This study has some limitations: the sample only included men aged 30, and only a few of them had cancer. Thus, more studies are needed to investigate this new technology. When it comes to breast cancer detection, the standard method for diagnosis is X-ray mammography (Muftuler et al., 2004). Hubbard et al. (2011) found that during a 10-year screening period, almost 7%–9% of patients received false-positive results. Additionally, research has shown that mammography misses 1 in 8 breast cancers in patients, and Nelson et al. (2016) found that this number increases in female patients aged between 40 and 79 years. A complete EIT system can complement current screening protocols by providing a compact and portable device (Wu et al., 2021). To elaborate on the previously mentioned technology, Lee et al. (2020) developed a 9.6 mW/CH 10 MHz wide-band EIT IC for breast cancer detection. Researchers tested their model on a breast cancer phantom constructed with carrots and agar and obtained high-precision images of the phantom with small phase errors of 1.2° during reconstruction. Additionally, the prototype allowed for successful preliminary detection of breast cancer (Lee et al., 2020). Akhtari-Zavare and Latiff (2015) tested the effectiveness of the electrical impedance computerized mammography (MEIK), an EIT system for breast cancer detection. The study was conducted on 88 patients with different breast complaints. MEIK results were compared to mammography (MG) and ultrasonography (USG) techniques. The authors found no difference in the sensitivity of detection between EIT, MG, and USG (Akhtari-Zavare and Latiff, 2015). Rotational EIT (rEIT) is described and shown to produce spatially accurate absolute reconstructions with improved image contrast and an improved ability to distinguish closely spaced inclusions compared to EIT. Murphy et al. (2016) explored rEIT for breast cancer imaging. To perform the experiment, researchers used a breast-shaped tank made of saline solution to simulate experimental conditions. Researchers found that absolute imaging for breast cancer can be achieved using rEIT. Additionally, extensive research is being conducted to apply this method to prostate cancer diagnosis (Murphy et al., 2016). Studies show that the electrical impedance of malignant tissues could be 20 to 40 times lower than that of healthy tissues. MREIT relies on a pre-existing MRI scanner, a constant current source, and a reconstruction algorithm (Woo and Seo, 2008). When biological materials are exposed to an external electric field, changes in their electrical properties become a source of magnetic field disturbances, which can be detected by the MR. Muftuler et al. (2004) investigated the clarity of the image of the tumor location using MREIT. The results showed that using MREIT, malignant tumors, specifically breast cancer, were identified with good spatial resolution. Temporary brachytherapy, known as HDR, is a radiation procedure treatment for prostate cancer. To maximize its performance and target the epicenter, an imaging technique is added to the procedure. Tan and Rossa (2021) presented EIT as the assisting imaging technique of choice. To assess the performance of EIT, the researchers tried detecting a large aluminum cylinder in different media. EIT was able to detect the cylinder in media of distilled water, sodium chloride, and gelatin, which were used to mimic human tissues. They were able to successfully identify bovine meat and chicken meat placed at different depths inside the medium. They concluded that using EIT, prostate cancer can be successfully detected, and HDR was proven to be an effective radiation treatment for it (Tan and Rossa, 2021).

## 6 Electrical impedance myography

Muscle tissue properties undergo changes due to factors such as gender, activity level, aging, and neuromuscular diseases. While exercise can strengthen muscles, aging can decrease their number and size (Distefano and Goodpaster, 2018). Neuromuscular diseases can cause muscle weakness and loss of muscle mass (Iolascon et al., 2019), which can also affect muscle conductivity and impedance. These changes can be easily detected through the use of EIM. Nescolarde et al. (2014) successfully used EIM to classify lower limb muscle injury among football athletes. Similarly, Kortman et al. (2013) used EIM measurements to group participants into categories based on factors such as gender, age, and sex. On the other hand, the portability of EIM made it a potential candidate for wearable devices and prosthetics (Wu et al., 2018).

### 6.1 EIM for prosthetics

The development of new ways to improve prosthetic control has been a topic of great interest in recent years. While EMG has traditionally been the most common approach for prosthetic control, recent research has explored an alternative approach using EIM. Cho et al. (2020) conducted an experiment to test whether the EIM signal could be used to control robotic hand prosthetics. The experiment involved attaching four-channel electrodes to the arms of 10 healthy men aged between 24 and 31 and repeating a set of 7 patterns that included movements such as flexion, extension, hand close, hand open, pronation, supination, and rest. The data collected from the experiment showed that EIM can be used for upper limb prostheses as the accuracy of the acquired data was similar to that obtained from an experiment using an EMG signal. However, it is important to note that EIM has some limitations as it is a signal that includes impedance from skin and fat, making it non-specific to muscle-tissue impedance. Additionally, EIM is sensitive to the movement of other body parts (Cho et al., 2020).

Hahne et al. (2021) also highlighted this characteristic of the EIM signal, noting that it is affected by any change in the arm position. They conducted a similar experiment to the one by Cho et al. (2020), performing a series of hand motions at three different positions. The results showed that the combination of EMG and EIM can successfully imitate the required movement of the arm as the EIM signal provides complementary information to the EMG signal. Ngo et al. (2022) also explored the idea of combining both EIM and EMG signals as this approach provides stronger measurements of the muscle’s condition. However, their study focused on developing a device to evaluate the muscle’s condition, force, and torque, rather than using it for prosthetic control.

Vavrinskỳ et al. (2018) focused on combining different signals, including EMG, mechanomyography (MMG), and EIM, to monitor muscular activities. Their experiments showed that EIM can be used as a means for robotics or prosthetic control as it is sensitive to isotonic signals and insensitive to isometric signals. Son et al. (2018) conducted three experiments to detect muscular activation with eight healthy participants aged between 23 and 29. The participants had electrodes wrapped around their forearms and went through an isometric muscle experiment, an isotonic experiment, and a frequency response experiment. The results showed that the EIM procedure can be directed toward prosthetic or exoskeleton robots for reading muscle action (Son et al., 2018). In another important finding, Cho et al. (2020) designed a robotic prosthetic hand that estimates kinematic changes in human hand states based on the EIM signal. Their design is invulnerable to common noise and external noise sources as it functions at a completely different frequency. This means that extensive filtering is not required, as in previous studies (Cho et al., 2020).

Recently, Cho et al. (2022) developed a robotic hand prosthetic named MSC hand. This prosthetic features multiple grasping speeds and a grasping force of 45N and is controlled by surface electromyographic signals. The movements of each finger are controlled by taking the signal of the corresponding electrode and processing it through a learning-based network that predicts how the finger should move. This study is an improvement over the one by Hahne et al. (2021) as the robotic hand now features a 3-degree of freedom (DOF) model, instead of a 2-DOF model. However, the study has a major limitation, in that it was only tested on healthy participants with fully intact arms. Since the characteristics of the EMG signal change in time and frequency after hand amputation, the study was unable to fully verify the functionality of the design for amputees (Hahne et al., 2021).

### 6.2 EIM for neuromuscular diseases

EIM is a technique that has been used for years to assess age-related diseases such as sarcopenia, which is the involuntary loss of skeletal muscle mass and strength and is considered a disease of the elderly (Santilli et al., 2014). As muscle function regresses with age, it is crucial to monitor this regression using clinically practical techniques that are easy to use. EIM has been studied for about two decades and has proven efficient in monitoring muscle health in a variety of conditions, including neuromuscular diseases, making it a practical tool to evaluate studies of aging.

Clark et al. (2021) conducted a study focused on proving the benefits of using EIM to monitor muscle health by demonstrating its sensitivity to microscopic changes as well as the loss of muscle function. To get their results, they conducted an experiment in which an electrical current was applied via two outer surface electrodes, and the voltage was measured via two inner electrodes across a healthy muscle in comparison to a sarcopenic muscle. This study highlights the different parameters presented by the EIM measurements, further validating the benefits of using this technique to assess changes in muscular properties affected by age (Clark et al., 2021).

EIM has also shown its sensitivity in measuring different muscular diseases, such as Duchenne muscular dystrophy (DMD), a condition that affects boys between the ages of 2–4 years. DMD symptoms begin with subtle weakened lower extremities that develop into the inability to walk. This condition affects nearly 1 in 3,000 male births. Rutkove and Darras (2013) used EIM measurements along with US for DMD patients and showed that EIM measurements can differentiate between healthy and diseased children while including age as a factor that affects the results. Moreover, EIM measurements were compared to MRI measurements, where the results were similar, categorizing EIM as a technology that surpasses standard clinical measures. Furthermore, other studies have also addressed age-related topics using EIM. Aaron et al. (2006) showed that EIM measurements differ with age. The conclusion was based on an experiment comprising 100 healthy individuals aged between 18 and 90 years, where EIM was used to measure across the quadriceps and the tibialis anterior of the individuals. The outcome of this study reflected a “quadratic reduction,” having a steeper declining curve for patients over 60 years old. The study also highlights the gender role, where the correlation was stronger in men (Aaron et al., 2006).

Likewise, Kortman et al. (2013) accentuated the changes in EIM with age while factoring in the effects of gender on the measurements targeting skeletal muscles. It has been proven that men face a clearer reduction in “lower extremity values” than women. Kortman et al. (2013) also emphasized the role of age when dealing with upper and lower extremity muscles. The experiments showed a higher reduction of the lower extremity muscles than the upper extremity ones. In addition, more recent studies have also focused on using EIM measurements to evaluate muscle changes. Arnold et al. (2017) and Hobson-Webb et al. (2018) study the effects of using EIM to detect muscular changes with age. Their studies were focused on using EIM for other purposes, such as the surveillance of muscular properties in mice, which showed a difference compared to aged animals (Arnold et al., 2017). Hobson-Webb et al. (2018) further illustrated that EIM may represent a reliable method to discern muscular deficit in older patients. The conclusion was reached after experimenting on 27 older adults with an average age of 72 years, where the measurements were consistent and reliable.

The usage of EIM goes beyond acute diseases and can also be used in chronic diseases, such as CLBP, a multifactorial disease with a lifetime duration of 75%–84%. CLBP is caused by abnormal movement of spinal segments that leads to pain in the spinal section. EIM has become a popular tool in assessing chronic diseases, and Wang H. et al. (2021) aimed to assess the electrical properties of the LPM in patients with CLBP and control patients. The results of the study showed that patients with CLBP had increased Z and R values and decreased PA values, while the X values were similar between the different test groups. Lukaski (2013) found that the size of the back muscle affected EIM measurements. This study had some limitations, such as the small sample size with a huge difference in age between the subjects, in addition to the anatomical structure of the lumbar crest, which did not allow EIM scanning in the transverse direction. Researchers concluded that the properties of LPM differed between CLBP patients and healthy subjects, indicating that patients with CLBP have fewer muscle fibers and an increase in fatty infiltration and connective tissue formation (Wang H. et al., 2021).

In an earlier study conducted by Wang et al. (2019), EIM was used to detect CLBP in 133 patients, 47 with CLBP and 86 healthy subjects. Researchers concluded that EIM may be helpful in the evaluation of patients with LBP, as the three main impedance variables, namely, resistivity, impedance, and phase, showed unique values and behaviors between healthy subjects and patients with LBP. Li et al. (2016) conducted a study on 19 patients to assess the changes in EIM at different levels of biceps brachii contractions during exhaustive exercise. The results showed a significant increase in resistance during high levels of contractions and a decrease in resistance during fatigue. The study also found a 60% increase in resistivity between a muscle at rest and a contracted one, suggesting that R can describe the architecture and metabolic figure of a muscle (Li et al., 2016). Shiffman et al. (2003) suggested that these changes might be related to the twofold phenomenon. Li et al. (2016) also found obvious fatigue in their test subjects, assessed through the accumulation of metabolites and intracellular fluids, as well as low resistance values. Additionally, researchers found that at 100 kHz, lower resistance and reactance were observed when compared to 50 kHz because the reactance and impedance of the membrane depend on the frequency of the current applied (Li et al., 2016).

Esper et al. (2006) examined the use of EIM in assessing amyotrophic lateral sclerosis (ALS) and inflammatory myopathy (IM), two commonly known chronic diseases. The experiment consisted of ALS and IM patients, as well as normal subjects, with a total of 6 men and 5 women being ALS patients, 5 women and 2 men being IM patients, and 46 normal subjects, consisting of 24 women and 22 men, all aged between 25 and 79 years. The goal of the study was to examine the multi-frequency patterns of EIM signals in patients compared to normal subjects. The study concluded that the multi-frequency EIM patterns varied among patients and normal subjects, but researchers were not able to differentiate between patients with myopathic and neurogenic diseases. However, researchers found that EIM signals could identify the severity of the disease (Esper et al., 2006).

EIM is a tool used to continuously monitor paralysis patients, with the most common cause being spinal cord injury (SCI). Li et al. (2017b) conducted two studies to assess the changes in electrical properties of muscles after SCI using EIM. The first study evaluated the biceps brachii of 17 patients with SCI and 23 control subjects. Researchers found a significant decrease in muscle reactance (X) and phase angle (θ) at frequencies of 50 kHz and 100 kHz in the SCI group. As a result, they concluded that EIM changes could be used to document changes in muscle in SCI patients as it requires no effort and is simple to conduct. In the second study, Li et al. (2017a) examined a new EIM technique to evaluate the hand muscles of 16 SCI patients and 18 control subjects. The thenar, hypothenar, and the first dorsal interosseous were evaluated, and the results were similar to those of the previous study. Researchers found lower reactance and phase angle in SCI patients than in the control group. They concluded that these changes in electrical properties could be due to changes in the membrane integrity and fat infiltration.

Hu et al. (2021) used myotonometry and EIM to evaluate the changes in muscle mechanical properties in patients with SCI. The study involved 36 subjects, 18 with SCI and 18 healthy subjects. Researchers evaluated the mechanical parameters of the muscle using the myotonometer, including oscillation, dynamic frequency, and stiffness. They also measured the electrical properties of the muscle using EIM, including reactance, resistivity, and phase angle. The results indicated a decrease in frequency, stiffness, and maxForce in the SCI group. Differences in R and $X_{C}$ were also observed between the SCI and control groups. Additionally, relaxation time and creep were significantly higher in the SCI group. Researchers observed a significant correlation between stiffness, maxForce, and $X_{C}$ . Their findings support the effectiveness of quantifying muscle mechanical and electric properties using EIM and myotonometers (Hu et al., 2021).

Although the scope of this paper focuses on human-centered applications, it is worth noting that EIM has also been applied to animals. For example, Kowal et al. (2022) evaluated EIM parameters in dogs with degenerative myelopathy (DM), revealing its effectiveness in distinguishing muscle changes and monitoring long-term disease progression. In advanced DM stages, EIM detected chronic low motor neuron signs. However, the study found no correlation between EIM phase values and gait scores, suggesting EIM’s limitations in identifying early-stage muscle diseases. Additionally, Hakim et al. (2017) demonstrated the utility of EIM in diagnosing muscle dystrophy in dogs. These findings underscore the potential of EIM beyond human applications although such animal studies fall outside the primary focus of this paper.

## 7 Electric cell-substrate impedance and applications in regenerative medicine and cardiology

Electric cell-substrate impedance (ECSi) has become a pivotal technology in assessing cellular behaviors and tissue interactions for regenerative medicine and cardiovascular applications. The development of ECSi has been accelerated by advances in both single- and multi-frequency impedance sensing, which allows detailed monitoring of cellular adhesion, proliferation, and interaction with conductive substrates, laying the groundwork for its use in complex tissue models (Li et al., 2024). Recent innovations, such as 3D bioprinting and biohybrid-engineered conductive patches, further underscore ECSi’s relevance in supporting and monitoring cell functionality in regenerative medicine, particularly for cardiovascular applications (Hwang et al., 2024).

### 7.1 Principles of electric cell-substrate impedance

ECSi operates by measuring impedance changes that occur as cells adhere to and interact with electrode arrays. These changes provide real-time insights into cellular properties, including adhesion strength and barrier function, without invasive labels. By coupling ECSi with high-density microelectrode arrays (HD-MEAs), researchers can track the spatial and temporal dynamics of cell behavior at a higher resolution. In particular, this technology has facilitated new studies on cardiomyocyte maturation and contractility under mechanical or electrical stimuli, which are key to advancing cardiac disease modeling and drug screening (Schmidt et al., 2024; Li et al., 2024).

### 7.2 Applications in cardiac regenerative medicine

The integration of ECSi with 3D bioprinting has facilitated the creation of physiologically relevant cardiac models. High-density microelectrode arrays, such as those developed for re-entry arrhythmia analysis, provide dual-modality monitoring of field potentials and contraction strength across large cardiac cell cultures. This allows for precise drug testing in hiPSC-derived cardiomyocytes and the evaluation of arrhythmogenic potential. Such setups enhance the predictivity of *in vitro* cardiac models, making them valuable for both regenerative research and translational applications (Schmidt et al., 2024; Hwang et al., 2024). Furthermore, engineered conductive cardiac patches, which incorporate advanced materials like platinum and graphene, have shown promise in post-infarction therapy. These patches facilitate cellular coupling and synchronous contraction in damaged cardiac tissues, demonstrating the regenerative potential of ECSi-based systems (Chen et al., 2024).

### 7.3 Engineered platforms for drug screening and disease modeling

ECSi is also instrumental in high-throughput drug screening within heart-on-a-chip platforms. Integrated with microfluidics and biosensors, these systems enable real-time monitoring of cellular responses to pharmacological agents by tracking contraction and electrophysiological stability in cardiomyocyte cultures. For instance, recent microfluidic devices employing ECSi and electrochemical impedance spectroscopy (EIS) have successfully mimicked human cardiac physiology, offering a predictive alternative to traditional animal models for cardiotoxicity testing (Dou et al., 2023; Criscione et al., 2023). Moreover, ECSi-based biosensors provide key information on dynamic cellular signals such as calcium flux and contractility, which are essential for evaluating both cardioprotective and regenerative drugs (Li et al., 2024).

## 8 Deep learning integration in electrical impedance analysis

Deep learning technology is rapidly evolving and being utilized in various industries, such as healthcare and medical technology. Deep learning in healthcare extends beyond data analysis and handling complex datasets. The technology is also being used in the radiology department through imaging and computer vision techniques. Additionally, deep learning is integrated into EIM, EIT, and EMG technologies to achieve improved image resolution and predict potential injuries. Many studies have been conducted to develop machine learning methods to assess and manage risk. These methods are evaluated by dividing the datasets into three categories: the training set, the validation set, and the testing set, which are acquired based on the specific technology used. Therefore, it is essential to obtain available datasets and examine how deep learning is used in conjunction with EIT and EIM to advance the understanding of this area (López-Valenciano et al., 2018).

### 8.1 Datasets available for EIT measurements

Across the literature, datasets have been collected for different purposes and used in various experiments. Significantly, EIDORS provides researchers with a wealth of resources to facilitate EIT research (Adler and Lionheart, 2005). Among these resources, EIDORS offers an extensive collection of tutorials and examples, spanning topics from fundamental image reconstruction to advanced techniques like dual models and unexpected effects. These tutorials not only empower researchers with knowledge but also provide practical guidance for implementing EIT effectively. Moreover, these tutorials can serve as valuable tools for generating custom datasets. For instance, researchers can utilize techniques such as the FEM demonstrated in these tutorials as a prime example of dataset generation. By gaining proficiency in EIT through EIDORS tutorials, researchers can apply their expertise to construct datasets using FEM, aligning datasets with their unique investigative needs. This multifaceted support ensures that EIDORS not only enhances researchers’ understanding of EIT but also equips them to create datasets that align precisely with their research goals.

Other datasets were collected by different research groups; one such dataset was collected by Jimbles and tdowrick (2018), which involves multi-frequency EIT measurements taken from 26 stroke patients. The aim was to create a dataset that could be used to develop EIT imaging methods specifically for stroke patients. The data were collected by placing 32 ECG electrodes and choosing a total of 31 injections, resulting in 992 measurements taken at 17 frequencies ranging between 5 Hz and 2 kHz. Jimbles and tdowrick (2018) later released an updated version of the dataset with structural improvements but with the same goal of collecting EIT data from stroke patients.

Another dataset focuses on the D-bar method, an image reconstruction method used in the EIT system to solve the inverse problem. This method is necessary since the reconstruction process is nonlinear. The purpose of this dataset is to present MATLAB routines for smooth symmetric conductivity (Mueller and Siltanen, 2012). A second dataset, also focused on the D-bar method, was developed by Mueller and Siltanen (2012) and includes MATLAB routines for a discontinuous heart-and-lungs phantom. Both datasets include computational MATLAB files taken from the same book.

Furthermore, a study conducted by Hauptmann et al. (2017) utilized a saline-filled tank to collect EIT measurements. The resulting dataset is divided into three parts: the first contains data on the current and voltage patterns of the tank with various targets, the second consists of photographs of the tank and targets, and the third is composed of MATLAB code for reading the data. EIT measurements were taken using stainless steel rectangular electrodes, with the tank filled to the top level of the electrodes. A total of 1,264 measurements were taken, resulting from 79 pairwise current injections and a voltage measurement between all adjacent electrodes for each injection. The publicly available datasets are summarized in Table 4.

**TABLE 4:** Summary of available datasets.

Data set	Data type	Who adopted it?	Citation
UCLH stroke EIT dataset patients-part 1	Measured	Cited by 52 different articles oriented toward multi-frequency EIT for stroke patients	Jimbles and tdowrick (2018)
EIT-team/stroke EIT dataset	Measured	Updated version of the original dataset It has 428 views and 80 downloads	Jimbles and tdowrick (2018)
EIT with the D-bar method: smooth and radical case	Synthetic	Cited by 551 other references with multiple topics ranging from a deep learning aspect to EIT and D-bar methods	Mueller and Siltanen (2012)
EIT with the D-bar method: discontinuous heart and lung phantom	Synthetic	Cited by 551 other references as the previous dataset	Mueller and Siltanen (2012)
Open 2D EIT data archive	Measured	Cited by 30 different articles where most of the articles were oriented toward the integration of EIT measurements with deep learning	Hauptmann et al. (2017)

### 8.2 Exploring deep learning applications in EIT

A primary challenge in EIT is the inverse problem of reconstructing conductivity distributions from boundary measurements, which is highly susceptible to noise and lacks uniqueness. Deep CNNs, such as the U-Net and attention-based models, have demonstrated strong performance in addressing these challenges. By leveraging convolutional layers to capture spatial features, CNNs enable the reconstruction of intricate tissue conductivity patterns and improve robustness against noise. For example, recent studies have employed attention-based deep CNNs, enhancing the reconstruction quality by focusing on critical regions within the impedance data, thus reducing artifacts and increasing the accuracy of image reconstruction (Yi et al., 2022; Wang et al., 2023).

The integration of deep learning techniques with EIT has garnered significant attention for its potential to overcome some of the inherent challenges associated with traditional EIT methods. Wei et al. (2019) focused on incorporating deep learning with EIT to address imaging challenges, particularly in recovering challenging inclusions like triangular, rectangular, or lung-shaped targets. They proposed two methods, an iterative-based inversion method and a CNN-based inversion method. The results showed that incorporating both methods with EIT improved the reconstruction of targets with sharp edges and corners. These approaches proved to be fast, stable, and capable of producing high-quality EIT imaging. Similarly, Ren et al. (2021) have also explored the integration of deep learning techniques into EIT, focusing on the dynamic image reconstruction challenges. They proposed a novel deep neural network framework, known as RCRC, to tackle the complexities associated with real-time conductivity reconstruction. This framework comprises a reconstruction network, a CNN encoder, a recurrent neural network (RNN) model, and a CNN decoder (thus referred to as RCRC), collectively designed to facilitate efficient filtering, smoothing, and prediction of dynamic conductivity reconstructions. The results of the experiment have successfully shown that RCRC can accurately recover dynamic conductivity images from EIT noisy voltage sequences (Ren et al., 2021).

In the context of image reconstruction in EIT, Li et al. (2021) proposed employing CNNs to address the challenges associated with image reconstruction. The motivation for utilizing CNNs in EIT stems from the inherent nonlinearity and under-qualification of the inverse problem, for which deep learning offers the capability of self-learning nonlinear mappings. The proposed method has yielded promising results. Ren et al. (2019) also highlighted the challenges posed by the inverse problem in EIT, particularly its impact on spatial resolution and modeling errors. Consequently, their study introduced a two-stage deep learning (TSDL) method, comprising a pre-reconstruction block and a CNN. The pre-reconstruction block is tailored to acquire regularization patterns from the training dataset, facilitating an initial target reconstruction. Subsequently, the CNN performs post-processing on the pre-reconstruction output, employing a multilevel feature analysis strategy to effectively mitigate modeling errors. The study’s outcomes underscore the TSDL method’s capacity to attain high-accuracy shape reconstructions and its robustness in the face of measurement noise and modeling errors.

The applications of EIT have also been highlighted in the manufacturing processes. However, the present challenges related to the inverse problem pose difficulties in real-time applications. To tackle these challenges, Aller et al. (2023) systematically compared six machine learning algorithms and investigated the impact of different EIT configurations. The findings of this research reveal that tree-based models, particularly gradient boosting, exhibit notable performance levels, surpassing even the commonly employed neural networks in handling EIT data. The study achieved an impressive 99.14% accuracy in detecting internal artifacts and a root mean square error of 4.75 in predicting internal conductivity distributions (Aller et al., 2023). Apart from using deep learning with EIT to improve image reconstruction, Smyl and Liu (2020) introduced a pragmatic approach based on deep learning for optimizing electrode positions. Achieving high-quality measurements necessitates a strategic placement of electrodes. The study’s findings reveal that the optimized electrode positions consistently outperform the conventional uniformly distributed layouts in all tested scenarios. Additionally, the use of these optimized positions leads to reduced errors in EIT reconstruction and enhances the discernibility of EIT measurements (Smyl and Liu, 2020).

The complexity of extending EIT from 2D to 3D further compounds the challenges associated with impedance imaging. To address this, researchers have explored learning-based 3D EIT reconstruction methods, such as transposed convolutional networks and graph neural networks. These models effectively handle the increase in data dimensionality, allowing for detailed 3D reconstructions essential for applications like lung and cardiac monitoring. In addition to spatial fidelity, these models provide robustness in heterogeneous conductivity scenarios, better reflecting realistic tissue properties than conventional methods (Tanyu et al., 2023).

### 8.3 Exploring deep learning applications in EIM

For EIM, DL models have been used to interpret muscle impedance data for applications in neuromuscular diagnostics and rehabilitation. Techniques such as RNNs and long short-term memory (LSTM) networks are particularly effective in analyzing time-series data, capturing temporal dependencies that are essential in monitoring muscle activity and fatigue over time. Additionally, DL models trained on extensive EIM datasets can differentiate between pathological and normal muscle states, providing valuable diagnostic insights and enabling real-time feedback in wearable EIM applications (Frerichs et al., 2023).

In recent years, the integration of deep learning with various technologies, such as EIM, has garnered significant attention. Pandeya et al. (2022) conducted a study focused on EIM as a “primary diagnostic technique” due to the combination of “multi-frequency resistance, reactance, and phase values.” The experiment involved measuring the impedance of four types of mice and wild-type animals, including 80 diseased mice and 33 WT animals, at different frequencies ranging from 8 to 1,027 KHz; this range was chosen to ensure that the measurements were not affected by electrode contact artifacts or inductive and parasitic capacitance artifacts. The range was determined to have a total of 29 frequencies, resulting in 174 values due to 29 frequencies with 2 electrode orientations and 3 datasets while having N = 113 samples.

Random forest (RF) was the algorithm of choice due to its ability to handle high-dimensional datasets. The results indicated that the approach of combining several features (“multi-frequency resistance, reactance, and phase values”) was better at classifying the diseased than a single frequency value (Pandeya et al., 2022). Moreover, EIM, a technology used to determine neurological diseases, has been utilized to classify different types of female breast tumors using machine learning techniques. Kabir and Ahad (2020) worked on this topic by classifying two breast tumor types, “benign and malignant,” using an ANN while having a 3D model of the female breast. The voltage was measured at a frequency of 500 kHz, and the EIM criteria, i.e., resistance (R), reactance (X), and phase (θ) , were extracted. A 3×1156 matrix was used for the input data, while a 2×1156 matrix was used for the target matrix, with the matrix operation carried out using the neural network toolbox of MATLAB v2019b. The results showed a 97.7% accuracy, proving the research theory that EIM parameters could be used as a classification technique to identify different types of breast tumors, even with varying breast shapes (Kabir and Ahad, 2020).

Furthermore, Srivastava et al. (2012) focused on disease assessment and used EIM with ML, along with quantitative muscle ultrasound (QMUS), to classify muscles with spinal muscular atrophy (SMA). An experiment was conducted on 46 participants, including 21 normal subjects, 15 SMA type 2 subjects, and 10 SMA type 3 subjects. The new model yielded higher area under the curve values than using each technology separately. Thus, ML allowed for the recognition of the difference between diseased (muscles having type 2 or type 3 SMA) and non-deceased muscles. In the same context, Alix et al. (2020) used EIM to evaluate the tongue muscles of patients with ALS by designing a system of two electrode plates placed on the patient’s tongue. They collected data from the EIM system for both ALS patients and healthy patients, comparing the results to validate the proposed system. The study used a machine learning approach to select the most relevant information from the results and reduce the dimension of the impedance data.

Likewise, Schooling et al. (2021) focused their research on using EIM to assess ALS. However, NTF was used in this study. NTF stands for non-negative tensor factorization and is an ML method that presents advantages that are relevant to the study at hand. Some of them are that it is capable of dealing with absent data, even though 70% of it would be missing. Furthermore, the use of NTF enables the output to be physically interpretable. Based on the conducted experiment involving healthy subjects and ASL patients, the hypothesis of the study was verified, which proved that NTF can successfully classify disease severity, allowing for clinical interpretations.

Cheng et al. (2022) employed EIM with machine learning to obtain the total mass of thigh muscles (MoTM), recording EIM parameters and subject characteristics such as age, weight, and BMI. They used ridge regression (RR) and support vector regression (SVR) to measure MoTM in 96 subjects and obtained better results than previous studies in terms of the regression coefficient and root mean square error (RMSE).

The application of EIM with machine learning is not limited to disease evaluation and diagnostic improvements. Barioul et al. (2020) explored the possibility of using this technological combination to identify a certain amount of American Sign Language (ASL). They recorded the change in impedance of the forearm due to several gestures and applied an extreme learning machine (ELM) classifier to data collected from 11 healthy subjects. The study found that a frequency range of 1 kHz–4 kHz was reliable for gesture recognition and resulted in an accuracy of 92.6% for training and validation data and an accuracy of 70.17% for testing data.

## 9 Discussion and recommendations

EIT and EIM are promising non-invasive techniques that have the potential to revolutionize healthcare applications. In this survey paper, we have reviewed the potential applications of EIT and EIM in healthcare and identified several areas of contribution to research. In addition, we have discussed the role of deep learning in advancing research in these fields.

Impedance devices generally face two main challenges. The first challenge is related to their use in clinical applications. Branco et al. (2023) tackled the use of bioelectrical impedance analysis (BIA) as a method to determine body composition for oncology patients. BIA consists of measuring the resistance (R) and reactance $\left(X_{c}\right)$ , which are used to determine the impedance (Z) . This impedance is then used to approximate the total body water (TBW). Thus, BIA allows estimating two main body composition models, the fat mass (FM) and the fat-free mass (FFM). By assessing bioelectric impedance vector analysis (BIVA)—which provides insights into hydration levels and cell mass—and phase angle (PhA), which reflects cell membrane integrity, valuable conclusions can be drawn about cancer-specific characteristics, such as its type and progression stage (Branco et al., 2023). However, the measurements taken by BIA are influenced by multiple clinical settings, leading to inconsistent results. These varied measurements are caused by the utilized measurement methodologies, patient preparation techniques, and a wide range of equipment used. The efficiency of BIA is also affected by individual factors such as limb length, menstrual cycle phase, physical activity, and nutritional condition. It was shown that the accuracy and precision of BIA measurements were poorer for obese/edematous individuals. Therefore, evaluations must be conducted under the same circumstances, taking into consideration possible sources of error (Branco et al., 2023).

The second challenge is related to the operating frequency of the impedance device. Padilha Leitzke and Zangl (2020) tackled the main difference between using EIT and electrical impedance tomography spectroscopy (EITS). EIT presents an ill-posed problem since it has difficulties using boundary measurements to achieve a single reliable solution. This leads to a difficult, accurate reconstruction of materials. Therefore, EITS is introduced as a multi-frequency approach that allows the differentiation of materials based on their frequency-dependent electrical properties. Through that approach, accurate material reconstruction is possible. The main difference between EIT and EITS is the multi-frequency approach since an EITS system operating at a single frequency is considered an EIT system, which reintroduces the ill-posed problem of EIT (Padilha Leitzke and Zangl, 2020).

Furthermore, Mansouri et al. (2021) discussed the difference between three different types of electrical impedance tomography, namely, conventional EIT, dual-frequency EIT, and multi-frequency EIT. Conventional EIT consists of using a single frequency (typically 50 kHz) to create an image based on two measurements taken at distinct times. However, it presents limitations related to reference stability, especially for long-term applications. Therefore, dual-frequency EIT is introduced to reduce the stability error presented by conventional EIT. This is done by applying currents at two distinct frequencies, which allows the distinction between tissue types and the detection of changes in conductivity related to the applied frequency. Multi-frequency EIT, on the other hand, consists of applying sinusoidal currents at different frequencies to get a more accurate tissue distinction since various tissue types react differently based on the frequency applied. Thus, through this method, errors presented in single-frequency systems are lowered (Mansouri et al., 2021).

One of the key areas of contribution to EIT research is the development of new imaging techniques. EIT has the potential to provide detailed images of the body’s internal structures and functions, and research in these fields could focus on developing new imaging techniques that are more accurate, sensitive, and specific. For example, researchers can use deep learning models to improve image segmentation and classification, which can lead to more accurate identification of abnormal tissues or structures. Furthermore, advances in sensor technology and data processing algorithms have the potential to improve the quality and resolution of EIT images, enabling clinicians to diagnose and monitor a wide range of health conditions more effectively.

Another important area of contribution is improving diagnostic capabilities. EIT and EIM can be used to diagnose a wide range of health conditions, including neuromuscular disorders, respiratory diseases, and tumors. Research in these fields could focus on developing new diagnostic tools and algorithms that can detect and diagnose diseases more accurately and efficiently. Machine learning and deep learning can be used to analyze large datasets and develop predictive models that can improve diagnosis accuracy, which can ultimately lead to better patient outcomes. For example, deep learning models can be used to classify and predict neuromuscular disorders based on EIM data, with promising results reported in recent studies (Shi et al., 2021).

Furthermore, EIT and EIM can be used to guide treatment planning and monitor the effectiveness of treatments. Research in these fields could focus on developing new algorithms and techniques that can optimize treatment planning and improve outcomes for patients. For example, EIT can be used to monitor lung function and optimize ventilation strategies in patients with respiratory failure, while EIM can be used to monitor muscle function and guide rehabilitation in patients with neuromuscular disorders (Pandeya et al., 2022). Machine learning and deep learning can be used to develop predictive models that can identify patterns in muscle activation and assess muscle function, which can help clinicians monitor disease progression and treatment effectiveness.

In addition, EIT and EIM have a wide range of potential applications in healthcare, and research in these fields could focus on investigating new applications and potential uses. For example, EIT can be used to monitor gastric function and detect gastrointestinal disorders, while EIM can be used to monitor cardiac function and detect early signs of heart disease. Machine learning and deep learning can be used to integrate data from multiple sources and develop comprehensive diagnostic tools that can detect and diagnose complex diseases.

EIT and EIM generate large amounts of data that require sophisticated analysis techniques. Research in these fields could focus on developing new data analysis methods that can extract meaningful information from the data more efficiently and accurately. Machine learning and deep learning can be used to analyze large datasets and identify meaningful patterns, which can ultimately improve our understanding of disease processes and treatment outcomes. For example, deep learning models can be used to predict patient outcomes based on EIM data, enabling clinicians to develop personalized treatment plans that are tailored to each patient’s unique needs.

The fields of EIT and EIM hold vast potential for contributions to healthcare applications. Machine learning and deep learning can be powerful tools in advancing research in these fields, enabling more accurate data analysis, prediction, and modeling. As such, researchers in these fields should continue to explore the potential of machine learning and deep learning techniques in improving the accuracy and efficiency of EIT and EIM imaging, diagnosis, treatment planning, and monitoring. Moreover, the collaboration between researchers in EIT and EIM, as well as experts in machine learning and deep learning, could facilitate the development of novel algorithms, tools, and techniques that can improve our understanding of the underlying biological processes and provide better healthcare solutions for patients. Furthermore, advancements in wearable technology present significant potential for the integration of EIT/EIM with deep learning in real-time monitoring for the patient’s status during everyday activities.

It is important to note that while EIT and EIM hold great promise, there are still several challenges that need to be addressed. For example, the complexity of the human body and the variability of physiological signals can lead to noise and artifacts in EIT and EIM data, which can affect the accuracy of image and data analysis. Additionally, the clinical translation of EIT and EIM techniques requires validation and standardization across different clinical settings and patient populations. These challenges highlight the need for further research in EIT and EIM and the potential of machine learning and deep learning to address these challenges and improve healthcare outcomes. The integration of reinforcement learning (RL) for optimizing impedance measurements is an emerging area in EIT and EIM. By dynamically adjusting measurement parameters, RL models adapt in real time to improve signal quality, thus enhancing diagnostic reliability in clinical environments. Additionally, the convergence of AI with real-time processing platforms is likely to expand impedance analysis capabilities in point-of-care diagnostics, supporting applications ranging from respiratory monitoring to neuromuscular health.

In summary, EIT and EIM are promising techniques with the potential to revolutionize healthcare applications. Machine learning and deep learning offer a powerful toolset to advance research in these fields and address some of the challenges associated with EIT and EIM. Continued research in EIT and EIM and collaboration between experts in these fields and in machine learning and deep learning can lead to the development of new imaging techniques, diagnostic tools, and treatment planning strategies that can improve healthcare outcomes for patients.

The main gaps in the literature and possible future research directions are listed below.

• Standardized protocols for BIA should be developed to improve consistency across clinical settings, considering patient-specific factors like physical condition, preparation, and measurement techniques.

• Multi-frequency EIT techniques, such as EITS, should be advanced to address the ill-posed nature of single-frequency EIT, improving material differentiation and accuracy in tissue reconstruction.

• Deep learning-based methods for EIT and EIM should be explored to enhance image segmentation, classification, and resolution, thereby improving accuracy in diagnosing and monitoring various health conditions.

• Robust diagnostic algorithms, potentially using deep learning models, should be developed to increase the predictive accuracy of neuromuscular, respiratory, and other disease diagnoses through EIT and EIM data analysis.

• Adaptive algorithms for treatment planning should be created, particularly using machine learning to tailor therapeutic strategies in real time, such as monitoring lung function for ventilation optimization or assessing muscle function during rehabilitation.

• Novel applications of EIT and EIM should be investigated in areas beyond current clinical uses, including the early detection of cardiac and gastrointestinal disorders and integration of multi-source data for more comprehensive diagnostic insights.

• Advanced data analysis techniques should be developed to handle the large datasets generated by EIT and EIM, incorporating machine learning to identify significant patterns that could improve understanding of disease progression and inform personalized treatment plans.

• Interdisciplinary collaboration should be promoted among researchers in EIT, EIM, and AI to design innovative tools and algorithms, addressing challenges in real-time data processing and increasing accuracy in complex physiological signal analysis.

• Research should be expanded into wearable technology to enable real-time monitoring of EIT and EIM data during daily activities, using machine learning to improve the integration of these techniques into wearable health monitoring systems.

• The noise and artifacts introduced by physiological variability in EIT and EIM measurements through advanced filtering and adaptive noise reduction techniques should be addressed.

• EIT and EIM across varied clinical settings and diverse patient demographics should be validated and standardized to ensure reliability and effectiveness in clinical translation.

• RL should be integrated into EIT and EIM systems for dynamic measurement optimization, allowing real-time adaptation to enhance signal quality and diagnostic reliability.

• The convergence of AI should be pursued with real-time processing technologies to expand EIT and EIM applications in point-of-care diagnostics, supporting conditions such as respiratory monitoring and neuromuscular health.

## 10 Conclusion

This review provides an overview of the two emerging electrical impedance technologies: myography and tomography. In this paper, we explain how injecting a current into the target tissue and recording the response at different frequencies can be used to obtain insightful information about the tissue—whether through constructing images or studying the tissue’s electrical properties. The review describes the basic components and methods involving these technologies while focusing on hardware parts, electrode selection, and electrode/injection configuration. We highlight key trends in the literature that serve as guidance to researchers who are interested in contributing to this field. For example, electrodes were used exclusively for either injection or recording in EIM, while in EIT, electrodes are used for both depending on the injection method used. The number of electrodes used increased with EIT, especially when high precision was required. Injection methods varied in quality when considering boundary conditions and the background noise, but all obtained impedance values were within the range. Ag/AgCl electrodes are most commonly used with all systems, and voltage injection was replaced by current injection due to the latter’s resistance to electrode wear out/drying and electrode impedance change upon long-term electrode use.

**FIGURE 10:**
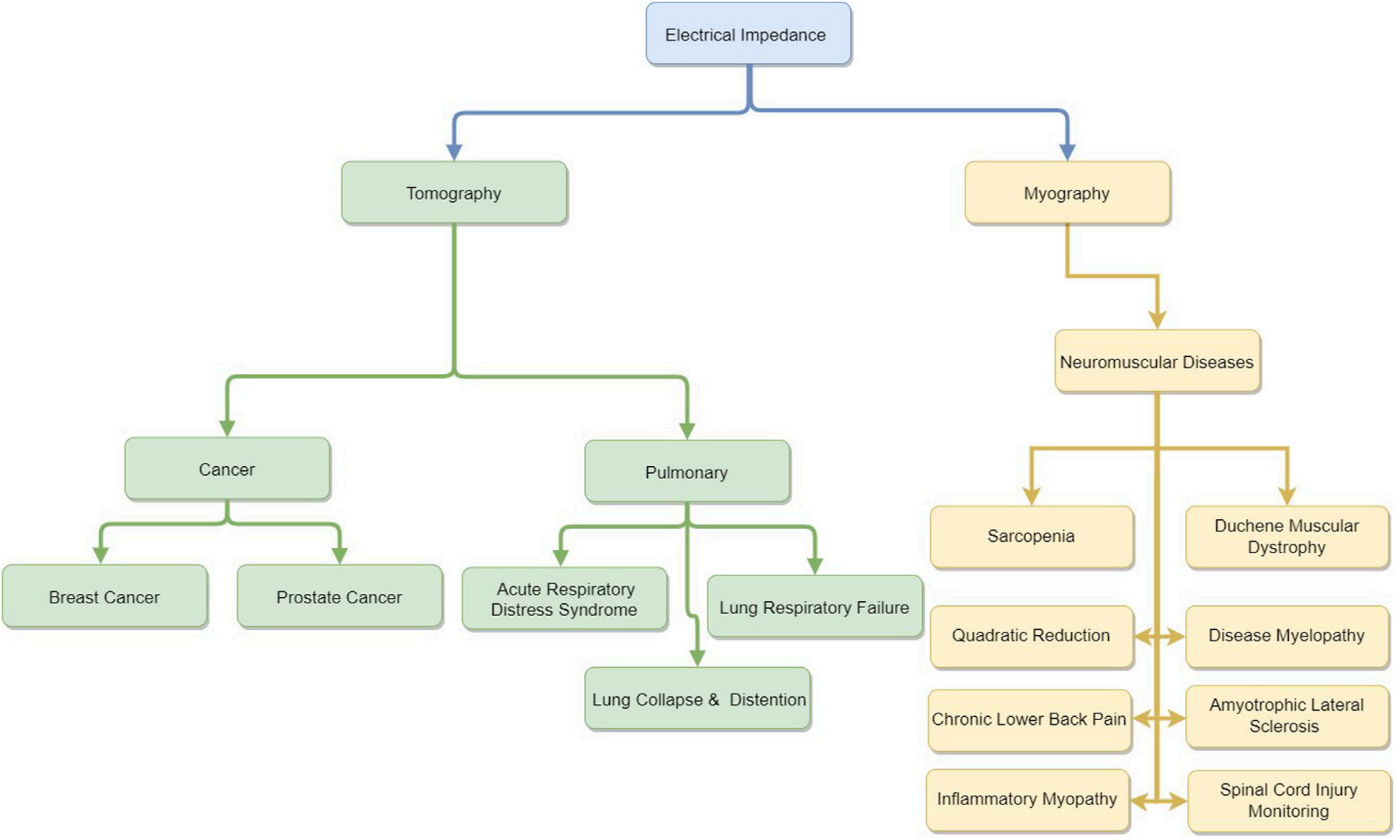
Use of EIM and EIT in disease diagnosis and clinical monitoring.

Furthermore, some trends have emerged alongside advances in fields such as digital processing and integrated circuits. A shift from analog to digital acquisition was observed in most EIM and EIT technologies, along with a transition from fixed regular current sources to voltage-controlled current sources. VCCSs are crucial in EIT and EIM systems as they allow incorporating several frequencies in the same device; thus, a VCCS with programmable and easily adjustable frequency and amplitude is preferred over regular constant sources. We also present the most powerful processing algorithms and reconstruction tools for both EIT and EIM while focusing on the strengths and weaknesses of each algorithm. Advances in EIM and EIT allowed the deployment of several devices in the market for individual and clinical use. We summarize commercial devices and present clinical uses for electrical impedance: EIT as a powerful imaging modality for detecting cancerous tissue and monitoring patients with pulmonary difficulties and EIM as a tool to detect and monitor the progress of neuromuscular diseases (Figure 10). Finally, we highlight the role of machine learning and deep learning in advancing the diagnosis, treatment planning, and monitoring of different diseases through the use of electrical impedance. Based on the findings of this review, the future of EIT/EIM-based research holds great potential. One of the viable research tracks is to integrate electrical impedance and deep learning in wearables to track the muscular activity of athletes and predict injuries. This presents a major turning point in sports on a medical and financial level.

This review charts a comprehensive guide to the field of impedance analysis. Research teams aiming to develop impedance devices will find valuable information on the evolution of these technologies, along with diagrams illustrating the fundamental components of any electrical impedance device. Those focused on impedance analysis will benefit from a comprehensive review of cutting-edge algorithms, reconstruction techniques, and available datasets. Moreover, clinicians and researchers integrating EIM and EIT into their studies will have access to a curated selection of commercial devices and an extensive body of previous clinical research, serving as a basis for potential future contributions.
