# Supplementary material for: Past, present, and future of electrical impedance tomography and myography for medical applications: a scoping review
Source: Front Bioeng Biotechnol. 2024 Dec 11;12:1486789. doi: 10.3389/fbioe.2024.1486789 (PMC11670078; doi:10.3389/fbioe.2024.1486789)
Supplement: Supplementary file 1 [file DataSheet1.pdf]

Supplementary Material

1 GLOSSARY

- **Acousto-Electric Impedance Tomography (aEIT):** A technique combining ultrasound with electrical impedance measurements to enhance imaging resolution and sensitivity for tissue properties, particularly used in soft tissue analysis.
- **Acute Respiratory Distress Syndrome (ARDS):** A severe lung condition causing fluid build-up in the alveoli, leading to decreased oxygen levels and difficulty breathing. ARDS often requires mechanical ventilation and intensive care.
- **American Sign Language (ASL):** A complete, natural language used by the Deaf community in the United States, utilizing hand shapes, facial expressions, and body movements to convey meaning.
- **Analog to Digital Converter (ADC):** An electronic device that converts continuous analog signals into discrete digital values, enabling digital processing of real-world signals.
- **Amyotrophic Lateral Sclerosis (ALS):** A progressive neurodegenerative disease that affects nerve cells in the brain and spinal cord, leading to the gradual loss of muscle control. ALS results in muscle weakness, paralysis, and, eventually, the inability to perform basic bodily functions. It is often known as Lou Gehrig's disease and currently has no cure.
- **Artificial Neural Network (ANN):** A computational model inspired by the structure and function of the human brain, consisting of interconnected layers of nodes (neurons) that process data. ANNs are commonly used in machine learning to recognize patterns, make predictions, and perform tasks such as classification and regression by learning from large datasets.
- **Bioelectric Impedance Vector Analysis (BIVA):** A method to assess body composition by analyzing impedance values, providing insights into hydration status, cell mass, and other physiological parameters.
- **Bioelectrical Impedance Analysis (BIA):** A technique used to estimate body composition, particularly fat and muscle mass, by measuring how electrical currents flow through body tissues.
- **Body Mass Index (BMI):** A measurement that evaluates an individual's body weight in relation to their height, calculated by dividing weight (in kilograms) by height (in meters squared).
- **Chronic Obstructive Lung Disease (COPD):** A chronic inflammatory lung disease that obstructs airflow from the lungs, often caused by smoking, resulting in breathing difficulties, cough, and reduced lung function.
- **Common Mode Rejection Ratio (CMRR):** A measure of a system's ability to reject common-mode signals, enhancing the accuracy of differential signal measurements by reducing interference.
- **Complete Electrode Model (CEM):** A detailed model used in Electrical Impedance Tomography (EIT) that includes both electrode impedance and contact impedance, providing accurate imaging.
- **Computed Tomography (CT):** A medical imaging technique that uses X-rays to create cross-sectional images of the body, providing detailed information on internal structures.
- **Convolutional Neural Network (CNN):** A deep learning model designed for processing structured grid data, commonly used in image and video analysis due to its ability to capture spatial hierarchies.

- **Degenerative Myelopathy (DM):** A progressive spinal cord disease in animals, particularly dogs, characterized by a gradual loss of motor function and mobility.
- **Degree of Freedom (DOF):** Refers to the number of independent movements allowed in a system, often used to describe robotic systems or physical models.
- **Digital Signal Processor (DSP):** A specialized microprocessor designed to perform high-speed mathematical operations essential for processing digital signals.
- **Digital to Analog Converter (DAC):** A device that converts digital signals back into analog form, commonly used in audio and video applications to reproduce real-world signals.
- **Duchenne Muscular Dystrophy (DMD):** A genetic disorder characterized by progressive muscle degeneration and weakness, primarily affecting boys, due to mutations in the dystrophin gene.
- **EIDORS (Electrical Impedance Tomography and Diffuse Optical Reconstruction Software):** An open-source software suite designed for image reconstruction in Electrical Impedance Tomography (EIT) and Diffuse Optical Tomography (DOT).
- **Electric Cell-Substrate Impedance (ECSi):** A technique for measuring the impedance of cells adhered to a substrate, providing insights into cell adhesion, growth, and behavior.
- **Electrical Impedance Computerized Mammograph (MEIK):** A diagnostic tool that combines electrical impedance measurements with mammography for improved breast tissue analysis.
- **Electrical Impedance Myography (EIM):** A non-invasive technique that assesses muscle health by measuring the electrical impedance properties of muscle tissue.
- **Electrical Impedance Spectroscopy (EIS):** A method for characterizing materials by measuring their impedance over a range of frequencies, often used in electrochemical and biological applications.
- **Electrical Impedance Tomography (EIT):** A non-invasive imaging technique that reconstructs cross-sectional images of electrical conductivity within a region of the body.
- **Electrical Impedance Tomography Spectroscopy (EITS):** An advanced form of EIT that acquires impedance data at multiple frequencies to improve tissue characterization.
- **Electromyography (EMG):** A diagnostic technique that measures the electrical activity produced by skeletal muscles during contraction and relaxation.
- **Extreme Learning Machine (ELM):** A type of feedforward neural network with a single hidden layer, known for its fast training speed and simplified structure.
- **Fat-Free Mass (FFM):** The total mass of all body components except fat, including muscles, bones, organs, and fluids, often measured to assess body composition.
- **Fat-Mass (FM):** The total weight of fat tissue in the body, an important metric for assessing body composition and health.
- **Field Programmable Gate Array (FPGA):** A type of integrated circuit that can be configured by the user after manufacturing, enabling custom digital circuitry.
- **Finite Element Method (FEM):** A numerical technique for solving complex physical problems by subdividing a large system into smaller, simpler parts, called finite elements.
- **Fish School Search Algorithm (FSS):** An optimization algorithm inspired by the collective behavior of fish schools, used to find solutions in large search spaces.
- **Genetic Algorithm (GA):** An optimization algorithm inspired by natural selection, used to find approximate solutions to complex problems by iteratively selecting and combining potential solutions.

- **Graz Consensus Reconstruction Algorithm for EIT (GREIT):** A standardized algorithm for image reconstruction in EIT, aiming to improve image quality and consistency across studies.
- **Gravitational Search Algorithm (GSA):** A metaheuristic optimization algorithm inspired by Newton's law of gravitation, where agents are attracted to each other based on fitness.
- **High-Density Microelectrode Arrays (HD-MEAs):** A device consisting of numerous small electrodes for recording electrical signals from cells, used in neuroscience and bioengineering research.
- **Impedance Plethysmography (IPG):** A technique for measuring changes in blood volume within an organ or tissue by detecting variations in electrical impedance.
- **Inflammatory Myopathy (IM):** A group of muscle diseases characterized by chronic muscle inflammation and weakness, often resulting from autoimmune conditions.
- **Limit of Agreement (LoA):** A statistical measure indicating the range within which two measurements agree, used to assess the reliability of different measurement methods.
- **Linearized Alternating Direction Method of Multipliers (LADMM):** An optimization algorithm that solves complex problems by decomposing them into simpler sub-problems and iterating.
- **Long Short-Term Memory (LSTM):** A type of Recurrent Neural Network (RNN) that uses gating mechanisms to retain long-term dependencies, ideal for sequence and time-series data.
- **Lumbar Paraspinal Muscles (LPM):** The muscles located beside the lumbar spine, providing support and stability to the lower back.
- **Magneto-Acousto Electric Impedance Tomography (MAET):** A hybrid imaging technique that combines magnetic, acoustic, and electrical impedance measurements for enhanced tissue characterization.
- **Magnetic Resonance Electrical Impedance Tomography (MREIT):** An imaging technique that combines MRI with EIT to map electrical conductivity distribution within tissues.
- **Magnetic Resonance Imaging (MRI):** A non-invasive imaging technique that uses powerful magnets, radio waves, and a computer to create detailed images of the body's internal structures.
- **Mammography (MG):** An imaging technique that uses low-dose X-rays to examine breast tissue, commonly used in screening for breast cancer.
- **Mass of Thigh Muscles:** The total weight or volume of muscle tissue in the thigh, including the quadriceps, hamstrings, and other surrounding muscles.
- **Mechanomyography (MMG):** A technique that measures the mechanical vibrations produced by muscle fibers during contraction, used to assess muscle function and fatigue.
- **Modified Newton Raphson Algorithm (MNR):** An iterative method for solving non-linear equations, commonly used in optimization and numerical analysis.
- **Newton's One-Step Error Reconstructor (NOSER):** A reconstruction algorithm used in EIT to produce images by iteratively minimizing error.
- **Non-negative Tensor Factorization (NTF):** A computational technique used to decompose high-dimensional, multi-way data (tensors) into a set of interpretable factors, with the constraint that all elements are non-negative.
- **Optical Projection Tomography (OPT):** An imaging technique that produces 3D reconstructions of small, transparent, or semi-transparent biological samples by capturing and combining multiple 2D images from different angles.

- **Positive End-Expiratory Pressure (PEEP):** A setting on a ventilator that maintains pressure in the lungs to prevent alveolar collapse at the end of exhalation, commonly used in respiratory therapy.
- **Prior Dual Interior-Point Methods (PDIPMs):** An optimization method used for solving linear and nonlinear programming problems, particularly effective for large-scale applications.
- **Quantitative Muscle Ultrasound (QMUS):** A diagnostic technique that uses ultrasound imaging to assess muscle structure, size, and composition quantitatively.
- **Random Forest:** A machine learning algorithm that operates as an ensemble of decision trees, combining the results of multiple trees to improve predictive accuracy and control overfitting.
- **Recurrent Neural Network (RNN):** A type of neural network that processes sequential data, such as time series or natural language, by maintaining information from previous steps.
- **Reinforcement Learning (RL):** A type of machine learning where an agent learns by interacting with an environment, receiving rewards or penalties, to maximize a long-term objective.
- **Ridge Regression (RR):** A linear regression technique that includes a regularization term, helping prevent overfitting by penalizing large coefficients.
- **Root Mean Square Error (RMSE):** A metric used to evaluate the accuracy of predictive models by measuring the average magnitude of the error between predicted and actual values.
- **Signal-to-Noise Ratio (SNR):** A measure of the strength of a signal relative to background noise, commonly used to assess data quality in communication systems and imaging.
- **Spinal Muscular Atrophy (SMA):** A genetic disorder characterized by the progressive loss of motor neurons, leading to muscle weakness and atrophy, with severity varying by type.
- **Support Vector Regression (SVR):** A type of Support Vector Machine (SVM) algorithm designed for regression tasks, aiming to find a function that fits within a margin of tolerance.
- **Total Body Water (TBW):** The amount of water contained within a human body, which includes intracellular and extracellular fluid compartments, used as a measure in hydration and health assessment.
- **Total Liquid Ventilation (TLV):** A ventilation technique where the lungs are filled with a breathable liquid, used in specialized medical procedures and experimental respiratory therapies.
- **Transrectal EIT (TREIT):** An application of Electrical Impedance Tomography via the rectum to produce cross-sectional images, commonly used in prostate and rectal examinations.
- **Transrectal Ultrasound (TRUS):** A diagnostic imaging technique where an ultrasound probe is inserted into the rectum to visualize the prostate and surrounding tissues.
- **Two-Stage Deep Learning (TSDL):** A deep learning framework where learning occurs in two stages, often involving pre-training and fine-tuning, to improve model accuracy and generalization.
- **Ultrasound (USG):** A non-invasive imaging technique that uses high-frequency sound waves to produce real-time images of the body's internal structures.
- **Ventilator-Induced Lung Injury (VILI):** Lung damage caused by mechanical ventilation, often resulting from excessive pressure or volume, highlighting the need for careful ventilation management.
- **Wild Type:** The typical form or natural phenotype of a species as it occurs in nature, often used as a reference in genetic and biological studies. Wild type represents the standard genotype or phenotype, with no mutations or alterations, against which genetically modified or mutated variants are compared.
